# Mathematical models of tissue stem and transit target cell divisions and the risk of radiation- or smoking-associated cancer

**DOI:** 10.1371/journal.pcbi.1005391

**Published:** 2017-02-14

**Authors:** Mark P. Little, Jolyon H. Hendry

**Affiliations:** 1 Radiation Epidemiology Branch, Division of Cancer Epidemiology and Genetics, National Cancer Institute, NIH, DHHS, Rockville, MD, United States of America; 2 Christie Medical Physics and Engineering, Christie Hospital and University of Manchester, Wilmslow Road, Manchester M20 4BX, United Kingdom; University of California Irvine, UNITED STATES

## Abstract

There is compelling biological data to suggest that cancer arises from a series of mutations in single target cells, resulting in defects in cell renewal and differentiation processes which lead to malignancy. Because much mutagenic damage is expressed following cell division, more-rapidly renewing tissues could be at higher risk because of the larger number of cell replications. Cairns suggested that renewing tissues may reduce cancer risk by partitioning the dividing cell populations into lineages comprising infrequently-dividing long-lived stem cells and frequently-dividing short-lived daughter transit cells. We develop generalizations of three recent cancer-induction models that account for the joint maintenance and renewal of stem and transit cells, also competing processes of partially transformed cell proliferation and differentiation/apoptosis. We are particularly interested in using these models to separately assess the probabilities of mutation and development of cancer associated with “spontaneous” processes and with those linked to a specific environmental mutagen, specifically ionizing radiation or cigarette smoking. All three models demonstrate substantial variation in cancer risks, by at least 20 orders of magnitude, depending on the assumed number of critical mutations required for cancer, and the stem-cell and transition-cell mutation rates. However, in most cases the conditional probabilities of cancer being mutagen-induced range between 7–96%. The relative risks associated with mutagen exposure compared to background rates are also stable, ranging from 1.0–16.0. Very few cancers, generally <0.5%, arise from mutations occurring solely in stem cells rather than in a combination of stem and transit cells. However, for cancers with 2 or 3 critical mutations, a substantial proportion of cancers, in some cases 100%, have at least one mutation derived from a mutated stem cell. Little difference is made to relative risks if competing processes of proliferation and differentiation in the partially transformed stem and transit cell population are allowed for, nor is any difference made if one assumes that transit cells require an extra mutation to confer malignancy from the number required by stem cells. The probability of a cancer being mutagen-induced correlates across cancer sites with the estimated cumulative number of stem cell divisions in the associated tissue (*p*<0.05), although in some cases there is sensitivity of findings to removal of high-leverage outliers and in some cases only modest variation in probability, but these issues do not affect the validity of the findings. There are no significant correlations (*p*>0.3) between lifetime cancer-site specific radiation risk and the probability of that cancer being mutagen-induced. These results do not depend on the assumed critical number of mutations leading to cancer, or on the assumed mutagen-associated mutation rate, within the generally-accepted ranges tested. However, there are borderline significant negative correlations (*p* = 0.08) between the smoking-associated mortality rate difference (current vs former smokers) and the probability of cancer being mutagen-induced. This is only the case where values of the critical number of mutations leading to cancer, *k*, is 3 or 4 and not for smaller values (1 or 2), but does not strongly depend on the assumed mutagen-associated mutation rate.

## Author summary

Cancer is thought to arise from a series of mutations in cells. Because mutations are expressed following cell division, more-rapidly renewing tissues could be at higher risk because of the larger number of divisions. Cairns suggested that tissues may reduce cancer risk by partitioning the dividing cell populations into lineages of infrequently-dividing stem cells and frequently-dividing daughter transit cells. We have developed generalizations of three recent cancer models that account for the joint maintenance and renewal of stem and transit cells, with particular focus on assessing the chance of cancer associated with radiation or smoking. All three models demonstrate substantial variation in cancer risks, spanning over twenty orders of magnitude. However, we show that if cancer occurs the chance that it is caused by a dominant mutagenic exposure is less variable, within an order of magnitude. Few cancers arise from mutations occurring solely in stem cells rather than in a combination of stem and transit cells. However, for cancers arising from 2–3 mutations, many have at least one mutation derived from a mutated stem cell. We confirm reports that the probability of a cancer being mutagen-induced is associated with the cumulative number of stem cell divisions in the relevant tissue.

## Introduction

As outlined by Harris [1] (see also ref. [2]), there are compelling biological data to suggest that cancer arises from a series of mutations which affect cell renewal and differentiation processes, and that cancer is largely unicellular in origin. Because much mutagenic damage is expressed following cell division, renewing tissues (e.g., the colon) may be at particular risk because of the large number of cell replications over a lifetime. Cairns [3] suggested that renewing tissues may have a lower risk of cancer than that based on total cell divisions of all cell types, by the separation of potential target-cell populations into long-lived stem cells and short-lived daughter transit cells. Cairns also suggested that there may be a segregation of old and new DNA strands, such that the old template strands were retained by the stem cells and the new strands with more errors were passed to the transit cells [3]. Stem cells are those cells in each tissue that are controlled by molecular signals and are responsible for maintaining tissue homeostasis. There is increasing attention devoted to stem cells and some of their differentiating daughter transit-cells, regarding their potential role as target cells in the carcinogenic process [4]. Mathematical models of stem and transit cell development have been constructed that are primarily based on the ideas of Cairns [3, 5], notably a model developed by Frank *et al*. [6].

A recent paper of Tomasetti and Vogelstein [7] aroused considerable interest, and suggested that “the lifetime risk of cancers of many different types [was] strongly correlated … with the total number of divisions of the normal self-renewing [stem] cells”. However, this interpretation has been challenged, in particular for the somewhat heterogeneous biological data used by Tomasetti and Vogelstein, being of rather variable quality [8]. Little *et al*. [9] subjected the data used by Tomasetti and Vogelstein [7] to detailed re-analysis, and concluded that they were “in conflict with predictions of a multistage model of carcinogenesis, under the assumption of homogeneity of numbers of driver mutations across most cancer sites”. Little *et al*. found no evidence for correlations between the extra-risk score developed by Tomasetti and Vogelstein and radiation- or smoking-related cancer risk [9]. Another reanalysis by Noble *et al*. of the data used by Tomasetti and Vogelstein [7], suggested that “cancer risk depends not only on the number of stem cell divisions but varies enormously (approx. 10 000 times) depending on anatomical site” [10]. A recent paper by Wu *et al*. [11] also re-analyzed the data of Tomasetti and Vogelstein [7]. Their analysis, combined with insights gained from a mathematical cancer model that they developed, suggested that “intrinsic [non-division-related] factors contribute only modestly (less than ~10–30% of lifetime risk) to cancer development”, a strikingly different assessment from that made by Tomasetti and Vogelstein [7]. The model of Wu *et al*. [11], which is a type of Galton-Watson branching process model [12], postulated a number of symmetric stem cell divisions until a given target stem cell number was attained, after which the stem cells divided asymmetrically, producing both further stem cells and non-stem transit cells. Cancer was assumed to arise if a given number of critical mutations arose, but cancer resulted only if these occurred within the stem cell population. This model [11] and the previous model of Frank *et al*. [6], yield mechanisms for stem and transit cell divisions that protect the critical stem cell population from excessive numbers of mutations, and yet still generate the necessary population of normal mature functional cells in each tissue.

In this paper we consider generalizations of the model of Frank *et al*. [6], and of Wu *et al*. [11], and a special case of a model developed by Little *et al*. [13]. We are particularly concerned with separately assessing the probabilities of mutation and development of cancer associated with “spontaneous” processes and with those linked to a specific dominant mutagen, namely ionizing radiation and smoking respectively, acting in addition to miscellaneous other endogenous and exogenous mutagenic processes.

## Methods

### Generalization of model of Frank *et al*. [6]

The model that we outline is somewhat similar to the model of Frank *et al*. [6], although more general. Frank *et al*. [6] did not derive the exact solution of their model, instead preferring various approximate solutions, which they compared with Monte Carlo simulations. Likewise we place most emphasis on a Monte Carlo implementation of a generalization of this model, in which cancer arises in a specific tissue if *k* critical “driver” mutations in particular genes are induced in a target cell. Such cells are assumed to arise from a stem cell that divides asymmetrically *n*_1_ times. At each division, the cell produces one daughter stem cell in which each critical gene is subject to mutation of the “spontaneous” (“intrinsic”) sort, with probability *u*_*S*,*s*_, or resulting from some specific mutagen, the “mutagen-induced” (“extrinsic”) sort, with probability *u*_*M*,*s*_. The division also produces one daughter transit cell in which each critical gene is subject to mutation from some specific dominant mutagen, the “mutagen-induced” (“extrinsic”) sort, with probability *u*_*M*,*t*_, or resulting from the “spontaneous” (“intrinsic”) sort associated with miscellaneous other endogenous and exogenous mutagenic processes, with probability *u*_*S*,*t*_. Each transit cell then undergoes *n*_2_ symmetric divisions. During each such division each critical gene in each daughter transit cell is subject to mutation of the “spontaneous” (“intrinsic”) sort, with probability *u*_*S*,*t*_, or resulting from some specific mutagen, the “mutagen-induced” (“extrinsic”) sort, with probability *u*_*M*,*t*_. Each of the mutational events taking place during stem cell division is statistically independent of the others, and assumed to be irreversible.

In the Monte Carlo implementation the model starts with a single stem cell. At the first cycle the stem cell divides into another stem cell and a single transit cell. At the second cycle the existing transit cell divides and the daughter stem cell divides into another stem cell and a daughter transit cell (resulting in a total of 3 transit cells). At the third cycle the 3 transit cells divide and the stem cell divides into another stem cell and a transit cell (resulting in a total of 7 transit cells). This carries on, until after *n*_1_th cycle all the stem cell divisions have taken place, and carries on for another *n*_2_ cycles until all transit cell divisions have also occurred. At the end of the tissue proliferation process, and at any intermediate stage, there is only a single stem cell. Implicit in this model is the idea that stem cell and transit cells have similar cycle times (but see Discussion). Potten and colleagues [14, 15] adduced evidence that the average cycle time of cycling cells (which would be mostly transit cells) is ~34 hours, and ≥36 hours for stem cells, in human colonic crypts.

The first cell in this division process that carries the *k* cancer mutations then is deemed to have caused cancer. The mutations can occur in any of the stem or transit lineages, and each transit cell derives its mutational burden initially from the particular generation of stem cell it came from. So if *k* = 3 cancer mutations in total are required, one could have a single mutation in a stem cell, and then two further transit cell mutations in the lineage derived from that stem cell (possibly via further stem cell divisions), or two mutations in the stem cell and a single mutation in a derived transit cell, or all three in a stem cell, or all three in a transit cell. The model can be easily generalized to the case in which the numbers of mutations required by stem and transit cells are different, as for example might be the case in the colon, as discussed by Frank *et al*. [6]. Such extensions of this model are not considered here, although we shall assess implications of a multi-stage cancer model that allows for this possibility (Table A6 in S1 Appendix).

**Fig 1:**
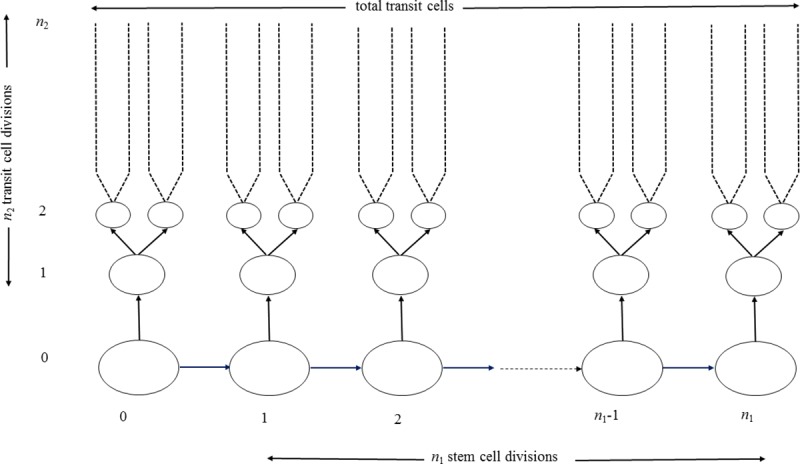
Schematic diagram of generalized stem cell model, corresponding to the model of Frank *et al*. [6]. The pattern of cell division gives rise to a total of *k* cells. The single initial stem cell divides to produce a stem cell lineage and a transit cell lineage. Each transit cell lineage divides *n*_2_ times yielding $2^{n_{2}}$ cells. The stem lineage divides *n*_1_ times, each division producing a daughter cell and a transit cell, thereby producing a total of $k=n_{1}2^{n_{2}}$ transit cells and a single stem cell.

This model is illustrated schematically in Fig 1. Therefore after *n*_1_ stem cell divisions and *n*_2_ transit cell divisions there is a single stem cell and $n_{1}2^{n_{2}}$ transit cells, so $n_{1}2^{n_{2}}+1$ of these cell types in total. We assume that these cell mutation rates can vary with numbers of cumulative cell divisions. We accumulate the numbers of each type of mutation in both stem and transit lineages. The first cell, whether a stem or a transit cell, that accumulates the necessary *k* critical cancer mutations is used to label the ensuing cancer that develops. We estimate the total probabilities of cancer, *C*_*tot*_, the probability of cancer that is due to at least one mutagen-associated mutation, *C*_*mut*_, the probability of cancer that arises from all mutations in the stem cell lineage, *C*_*stem*–*tot*_, and the probability of cancer that arises from at least one mutation in the stem cell lineage, *C*_*stem*–*part*_. These probabilities are then used to determine the conditional probability, given that cancer develops, that it is due to at least one mutation produced by the specified mutagen:

$${\mathrm{Pr}}_{M}=\frac{C_{mut}}{C_{tot}}$$ (1)

It may also be of interest to calculate the conditional probability, given that cancer develops, that it is due to the critical mutations developing entirely in the stem-cell lineage, given by:

$${\mathrm{Pr}}_{stem-tot}=\frac{C_{stem-tot}}{C_{tot}}$$ (2)

or that it is due to at least one of the critical mutations developing in the stem-cell lineage, given by:

$${\mathrm{Pr}}_{stem-part}=\frac{C_{stem-part}}{C_{tot}}$$ (3)

We also estimate the relative risk (RR), which is the ratio of the given total cancer probability, *C*_*tot*_, to the total cancer probability with the mutagen-associated mutation rates set to 0, *C*_*tot*,0_:

$$RR_{M}=\frac{C_{tot}}{C_{tot,0}}$$ (4)

We illustrate with calculations given in Table 1, using a spontaneous stem-cell mutation rate (*u*_*S*,*s*_) = 10^−8^, 10^−7^, 10^−6^, 10^−5^ or 10^−4^ per cell division, a spontaneous transition-cell mutation rate (*u*_*S*,*t*_) = 10^−6^, 10^−5^ or 10^−4^ per cell division, and *k* = 1 to 3 critical cancer genes. The mutagen-associated mutation rates for stem and transit cells are in the range of 0–100% of the spontaneous rates, and are assumed to apply over the last two thirds of cell division cycles (i.e., the last two thirds of the *n*_1_ + *n*_2_ cycles), the rates before that being 0. The scenario of spontaneous mutations increasing over the last two-thirds of all cell cycles corresponds roughly to a person being “unexposed” (to some specific mutagen) in early life, then “exposed” later in the process of development of some tissue. The “third” here is somewhat arbitrary, but would be consistent with occupational exposure to some mutagen, e.g., ionizing radiation, or exposure in adulthood for some other mutagen, e.g., cigarette smoke. The total (spontaneous + mutagen-associated) stem-cell and transition-cell mutation rates are therefore *u*_*s*_ = *u*_*S*,*s*_ + *u*_*M*,*s*_ = 10^−8^ to 2 x 10^−4^ per cell division and *u*_*t*_ = *u*_*S*,*t*_ + *u*_*M*,*t*_ = 10^−6^ to 2 x 10^−4^ per cell division, similar to those assumed by Frank *et al*. [6], which spanned the range *u*_*s*_ = 10^−10^ to 10^−5^ and *u*_*t*_ = 10^−6^ to 10^−3^ per cell division. So, for example, taking the third row in Table 1, the stem cell mutation rate is 1 x 10^−6^ and the transit cell mutation rate is 1 x 10^−4^ in the first third of the *n*_1_ + *n*_2_ cell cycles, then for the remaining two thirds of the *n*_1_ + *n*_2_ cycles the stem cell mutation rate increases to 1 x 10^−6^ + 5 x 10^−7^ = 1.5 x 10^−6^ and the transit cell mutation rate increases to 1 x 10^−4^ + 5 x 10^−5^ = 1.5 x 10^−4^. Frank *et al*. [6] assumed *k* = 2 critical cancer genes. We assume a number of asymmetric stem cell divisions *n*_1_ = 1024 and a number of symmetric transit cell divisions *n*_2_ = 10, corresponding to 2^20^ total stem and transit cells (*N* = 20). *n*_2_ = 10 was used as a generic order of magnitude, based on steady-state estimates of 5–9 transit cell divisions for colon, about 3 such divisions in epidermis, and 5–8 transit divisions in the various lineages in hematopoiesis [4].

**Table 1:** Probabilities of cancer being mutagen induced, evaluated using Monte Carlo implementation of a generalization of the stem cell model of Frank *et al*. [6], assuming spontaneous stem-cell mutation rate (*u*_*S*,*s*_) = 10^−8^, 10^−7^, 10^−6^, 10^−5^, or 10^−4^ per cell division, spontaneous transition-cell mutation rate (*u*_*S*,*t*_) = 10^−6^, 10^−5^, or 10^−4^ per cell division, and *k* = 1 to 3 critical cancer genes. **We assume a number of symmetric cell divisions *n***_**1**_ **= 1024 and a number of asymmetric cell divisions *n***_**2**_ **= 10, corresponding to 2**^**20**^ **total cells (*N* = 20), as in the paper of Frank *et al*. [6].** Unless otherwise stated all probability estimates are based on 10,000 Monte Carlo samples.

Cancer mutations *k*	*u*_*S*,*s*_	*u*_*M*,*s*_	*u*_*S*,*t*_	*u*_*M*,*t*_	Total probability of cancer	Total probability of mutagen-associated cancer	Pr[cancer mutagen associated| cancer develops]	Pr[all mutations in stem cells giving cancer]	Pr[all cancer mutations in stem-cell|cancer develops]	Pr[some mutations in stem cells giving cancer]	Pr [some mutations in stem cells|cancer develops]	Relative risk
1	1 x 10^−6^	0	1 x 10^−4^	0	1.0000	0	0	<0.0001	<0.0001	<0.0001	<0.0001	1.00
1	1 x 10^−6^	2 x 10^−7^	1 x 10^−4^	2 x 10^−5^	1.0000	<0.0001	<0.0001	<0.0001	<0.0001	<0.0001	<0.0001	1.00
1	1 x 10^−6^	5 x 10^−7^	1 x 10^−4^	5 x 10^−5^	1.0000	<0.0001	<0.0001	<0.0001	<0.0001	<0.0001	<0.0001	1.00
1	1 x 10^−6^	1 x 10^−6^	1 x 10^−4^	1 x 10^−4^	1.0000	<0.0001	<0.0001	<0.0001	<0.0001	<0.0001	<0.0001	1.00
1	1 x 10^−7^	0	1 x 10^−5^	0	1.0000	0	0	<0.0001	<0.0001	<0.0001	<0.0001	1.00
1	1 x 10^−7^	2 x 10^−8^	1 x 10^−5^	2 x 10^−6^	1.0000	0.0002	0.0002	<0.0001	<0.0001	<0.0001	<0.0001	1.00
1	1 x 10^−7^	5 x 10^−8^	1 x 10^−5^	5 x 10^−6^	1.0000	0.0001	0.0001	<0.0001	<0.0001	<0.0001	<0.0001	1.00
1	1 x 10^−7^	1 x 10^−7^	1 x 10^−5^	1 x 10^−5^	1.0000	0.0004	0.0004	<0.0001	<0.0001	<0.0001	<0.0001	1.00
1	1 x 10^−8^	0	1 x 10^−6^	0	0.8775	0	0	<0.0001	<0.0001	<0.0001	<0.0001	1.00
1	1 x 10^−8^	2 x 10^−9^	1 x 10^−6^	2 x 10^−7^	0.9074	0.0680	0.0749	<0.0001	<0.0001	<0.0001	<0.0001	1.03
1	1 x 10^−8^	5 x 10^−9^	1 x 10^−6^	5 x 10^−7^	0.9407	0.1496	0.1590	<0.0001	<0.0001	<0.0001	<0.0001	1.07
1	1 x 10^−8^	1 x 10^−8^	1 x 10^−6^	1 x 10^−6^	0.9725	0.2323	0.2389	<0.0001	<0.0001	<0.0001	<0.0001	1.11
2	1 x 10^−6^	0	1 x 10^−4^	0	0.3210	0	0	<0.0001	<0.0001	0.0013	0.0040	1.00
2	1 x 10^−6^	2 x 10^−7^	1 x 10^−4^	2 x 10^−5^	0.3919	0.0821	0.2095	<0.0001	<0.0001	0.0016	0.0041	1.22
2	1 x 10^−6^	5 x 10^−7^	1 x 10^−4^	5 x 10^−5^	0.5050	0.2173	0.4303	<0.0001	<0.0001	0.0024	0.0048	1.57
2	1 x 10^−6^	1 x 10^−6^	1 x 10^−4^	1 x 10^−4^	0.6867	0.4220	0.6145	<0.0001	<0.0001	0.0019	0.0028	2.14
2	1 x 10^−7^	0	1 x 10^−5^	0	0.0047	0	0	<0.0001	<0.0001	0.0001	0.0213	1.00
2	1 x 10^−7^	2 x 10^−8^	1 x 10^−5^	2 x 10^−6^	0.0052	0.0007	0.1346	<0.0001	<0.0001	<0.0001	<0.0001	1.11
2	1 x 10^−7^	5 x 10^−8^	1 x 10^−5^	5 x 10^−6^	0.0074	0.0030	0.4054	<0.0001	<0.0001	0.0001	0.0135	1.57
2	1 x 10^−7^	1 x 10^−7^	1 x 10^−5^	1 x 10^−5^	0.0119	0.0085	0.7143	<0.0001	<0.0001	0.0003	0.0252	2.53
2	1 x 10^−8^	0	1 x 10^−6^	0	<0.0001^a^	0	0	<0.0001^a^	NA^a^^,^ ^b^	<0.0001^a^	NA^a^^,^ ^b^	NA^a^^,^ ^b^
2	1 x 10^−8^	2 x 10^−9^	1 x 10^−6^	2 x 10^−7^	<0.0001^a^	<0.0001^a^	NA^a^^,^ ^b^	<0.0001^a^	NA^a^^,^ ^b^	<0.0001^a^	NA^a^^,^ ^b^	NA^a^^,^ ^b^
2	1 x 10^−8^	5 x 10^−9^	1 x 10^−6^	5 x 10^−7^	0.0001^a^	<0.0001^a^	0.3333^a^	<0.0001^a^	<0.0001^a^	<0.0001^a^	0.0833^a^	NA^a^^,^ ^b^
2	1 x 10^−8^	1 x 10^−8^	1 x 10^−6^	1 x 10^−6^	0.0001^a^	<0.0001^a^	0.5833^a^	<0.0001^a^	<0.0001^a^	<0.0001^a^	0.1667^a^	NA^a,^ ^b^
3	1 x 10^−6^	0	1 x 10^−4^	0	0.0012^a^	0	0	<0.0001^a^	<0.0001^a^	0.0006^a^	0.5250^a^	1.00^a^
3	1 x 10^−6^	2 x 10^−7^	1 x 10^−4^	2 x 10^−5^	0.0015^a^	0.0005^a^	0.3333^a^	<0.0001^a^	<0.0001^a^	0.0007^a^	0.4933^a^	1.25^a^
3	1 x 10^−6^	5 x 10^−7^	1 x 10^−4^	5 x 10^−5^	0.0031^a^	0.0019^a^	0.5987^a^	<0.0001^a^	<0.0001^a^	0.0015^a^	0.4725^a^	2.58^a^
3	1 x 10^−6^	1 x 10^−6^	1 x 10^−4^	1 x 10^−4^	0.0054^a^	0.0043^a^	0.7856^a^	<0.0001^a^	<0.0001^a^	0.0022^a^	0.4011^a^	4.51^a^
3	1 x 10^−7^	0	1 x 10^−5^	0	<0.0001^a^	0	0	<0.0001^a^	NA^a^^,^ ^b^	<0.0001^a^	NA^a^^,^ ^b^	NA^a^^,^ ^b^
3	1 x 10^−7^	2 x 10^−8^	1 x 10^−5^	2 x 10^−6^	<0.0001^a^	<0.0001^a^	NA^a^^,^ ^b^	<0.0001^a^	NA^a^^,^ ^b^	<0.0001^a^	NA^a^^,^ ^b^	NA^a^^,^ ^b^
3	1 x 10^−7^	5 x 10^−8^	1 x 10^−5^	5 x 10^−6^	<0.0001^a^	<0.0001^a^	NA^a^^,^ ^b^	<0.0001^a^	NA^a^^,^ ^b^	<0.0001^a^	NA^a^^,^ ^b^	NA^a^^,^ ^b^
3	1 x 10^−7^	1 x 10^−7^	1 x 10^−5^	1 x 10^−5^	<0.0001^a^	<0.0001^a^	NA^a^^,^ ^b^	<0.0001^a^	NA^a^^,^ ^b^	<0.0001^a^	NA^a^^,^ ^b^	NA^a^^,^ ^b^
3	1 x 10^−8^	0	1 x 10^−6^	0	<0.0001^a^	0	0	<0.0001^a^	NA^a^^,^ ^b^	<0.0001^a^	NA^a^^,^ ^b^	NA^a^^,^ ^b^
3	1 x 10^−8^	2 x 10^−9^	1 x 10^−6^	2 x 10^−7^	<0.0001^a^	<0.0001^a^	NA^a^^,^ ^b^	<0.0001^a^	NA^a^^,^ ^b^	<0.0001^a^	NA^a^^,^ ^b^	NA^a^^,^ ^b^
3	1 x 10^−8^	5 x 10^−9^	1 x 10^−6^	5 x 10^−7^	<0.0001^a^	<0.0001^a^	NA^a^^,^ ^b^	<0.0001^a^	NA^a^^,^ ^b^	<0.0001^a^	NA^a^^,^ ^b^	NA^a^^,^ ^b^
3	1 x 10^−8^	1 x 10^−8^	1 x 10^−6^	1 x 10^−6^	<0.0001^a^	<0.0001^a^	NA^a^^,^ ^b^	<0.0001^a^	NA^a^^,^ ^b^	<0.0001^a^	NA^a^^,^ ^b^	NA^a^^,^ ^b^
1	1 x 10^−4^	0	1 x 10^−4^	0	1.0000	0	0	0.0012	0.0012	0.0012	0.0012	1.00
1	1 x 10^−4^	2 x 10^−5^	1 x 10^−4^	2 x 10^−5^	1.0000	<0.0001	<0.0001	0.0012	0.0012	0.0012	0.0012	1.00
1	1 x 10^−4^	5 x 10^−5^	1 x 10^−4^	5 x 10^−5^	1.0000	<0.0001	<0.0001	0.0012	0.0012	0.0012	0.0012	1.00
1	1 x 10^−4^	1 x 10^−4^	1 x 10^−4^	1 x 10^−4^	1.0000	<0.0001	<0.0001	0.0012	0.0012	0.0012	0.0012	1.00
1	1 x 10^−5^	0	1 x 10^−5^	0	1.0000	0	0	0.0005	0.0005	0.0005	0.0005	1.00
1	1 x 10^−5^	2 x 10^−6^	1 x 10^−5^	2 x 10^−6^	1.0000	0.0002	0.0002	0.0005	0.0005	0.0005	0.0005	1.00
1	1 x 10^−5^	5 x 10^−6^	1 x 10^−5^	5 x 10^−6^	1.0000	0.0001	0.0001	0.0005	0.0005	0.0005	0.0005	1.00
1	1 x 10^−5^	1 x 10^−5^	1 x 10^−5^	1 x 10^−5^	1.0000	0.0004	0.0004	0.0005	0.0005	0.0005	0.0005	1.00
1	1 x 10^−6^	0	1 x 10^−6^	0	0.8777	0	0	0.0006	0.0007	0.0006	0.0007	1.00
1	1 x 10^−6^	2 x 10^−7^	1 x 10^−6^	2 x 10^−7^	0.9074	0.0681	0.0750	0.0005	0.0006	0.0005	0.0006	1.03
1	1 x 10^−6^	5 x 10^−7^	1 x 10^−6^	5 x 10^−7^	0.9408	0.1493	0.1587	0.0005	0.0005	0.0005	0.0005	1.07
1	1 x 10^−6^	1 x 10^−6^	1 x 10^−6^	1 x 10^−6^	0.9725	0.2322	0.2388	0.0005	0.0005	0.0005	0.0005	1.11
2	1 x 10^−4^	0	1 x 10^−4^	0	0.4464	0	0	0.0002	0.0004	0.1564	0.3504	1.00
2	1 x 10^−4^	2 x 10^−5^	1 x 10^−4^	2 x 10^−5^	0.5180	0.1068	0.2062	0.0002	0.0004	0.1630	0.3147	1.16
2	1 x 10^−4^	5 x 10^−5^	1 x 10^−4^	5 x 10^−5^	0.6195	0.2411	0.3892	0.0005	0.0008	0.1789	0.2888	1.39
2	1 x 10^−4^	1 x 10^−4^	1 x 10^−4^	1 x 10^−4^	0.7722	0.4521	0.5855	0.0004	0.0005	0.1869	0.2420	1.73
2	1 x 10^−5^	0	1 x 10^−5^	0	0.0213	0	0	<0.0001	<0.0001	0.0168	0.7887	1.00
2	1 x 10^−5^	2 x 10^−6^	1 x 10^−5^	2 x 10^−6^	0.0298	0.0075	0.2517	<0.0001	<0.0001	0.0231	0.7752	1.40
2	1 x 10^−5^	5 x 10^−6^	1 x 10^−5^	5 x 10^−6^	0.0323	0.0142	0.4396	<0.0001	<0.0001	0.0263	0.8142	1.52
2	1 x 10^−5^	1 x 10^−5^	1 x 10^−5^	1 x 10^−5^	0.0476	0.0320	0.6723	0.0001	0.0021	0.0371	0.7794	2.23
2	1 x 10^−6^	0	1 x 10^−6^	0	0.0010	0	0	<0.0001	<0.0001	0.0010	1.0000	1.00
2	1 x 10^−6^	2 x 10^−7^	1 x 10^−6^	2 x 10^−7^	0.0013	0.0001	0.0769	<0.0001	<0.0001	0.0013	1.0000	1.30
2	1 x 10^−6^	5 x 10^−7^	1 x 10^−6^	5 x 10^−7^	0.0016	0.0008	0.5000	<0.0001	<0.0001	0.0016	1.0000	1.60
2	1 x 10^−6^	1 x 10^−6^	1 x 10^−6^	1 x 10^−6^	0.0028	0.0019	0.6786	<0.0001	<0.0001	0.0025	0.8929	2.80
3	1 x 10^−4^	0	1 x 10^−4^	0	0.0698^a^	0	0	<0.0001^a^	0.0007^a^	0.0693^a^	0.9921^a^	1.00^a^
3	1 x 10^−4^	2 x 10^−5^	1 x 10^−4^	2 x 10^−5^	0.0924^a^	0.0312^a^	0.3378^a^	<0.0001^a^	0.0003^a^	0.0916^a^	0.9916^a^	1.32^a^
3	1 x 10^−4^	5 x 10^−5^	1 x 10^−4^	5 x 10^−5^	0.1361^a^	0.0815^a^	0.5989^a^	<0.0001^a^	0.0002^a^	0.1349^a^	0.9905^a^	1.95^a^
3	1 x 10^−4^	1 x 10^−4^	1 x 10^−4^	1 x 10^−4^	0.2151^a^	0.1722^a^	0.8008^a^	0.0002^a^	0.0009^a^	0.2126^a^	0.9883^a^	3.08^a^
3	1 x 10^−5^	0	1 x 10^−5^	0	0.0004^a^	0	0	<0.0001^a^	<0.0001^a^	0.0004^a^	1.0000^a^	1.00^a^
3	1 x 10^−5^	2 x 10^−6^	1 x 10^−5^	2 x 10^−6^	0.0005^a^	0.0002^a^	0.3922^a^	<0.0001^a^	<0.0001^a^	0.0005^a^	1.0000^a^	1.34^a^
3	1 x 10^−5^	5 x 10^−6^	1 x 10^−5^	5 x 10^−6^	0.0007^a^	0.0004^a^	0.5942^a^	<0.0001^a^	<0.0001^a^	0.0007^a^	1.0000^a^	1.82^a^
3	1 x 10^−5^	1 x 10^−5^	1 x 10^−5^	1 x 10^−5^	0.0013^a^	0.0010^a^	0.7907^a^	<0.0001^a^	<0.0001^a^	0.0013^a^	1.0000^a^	3.39^a^
3	1 x 10^−6^	0	1 x 10^−6^	0	<0.0001^a^	0	0	<0.0001^a^	NA^a^^,^ ^b^	<0.0001^a^	NA^a^^,^ ^b^	NA^a^
3	1 x 10^−6^	2 x 10^−7^	1 x 10^−6^	2 x 10^−7^	<0.0001^a^	<0.0001^a^	NA^a^^,^ ^b^	<0.0001^a^	NA^a^^,^ ^b^	<0.0001^a^	NA^a^^,^ ^b^	NA^a^
3	1 x 10^−6^	5 x 10^−7^	1 x 10^−6^	5 x 10^−7^	<0.0001^a^	<0.0001^a^	NA^a^^,^ ^b^	<0.0001^a^	NA^a^^,^ ^b^	<0.0001^a^	NA^a^^,^ ^b^	NA^a^
3	1 x 10^−6^	1 x 10^−6^	1 x 10^−6^	1 x 10^−6^	<0.0001^a^	<0.0001^a^	NA^a^^,^ ^b^	<0.0001^a^	NA^a^^,^ ^b^	<0.0001^a^	NA^a^^,^ ^b^	NA^a^

^a^simulation based on 100,000 Monte Carlo samples.

^b^some component Monte-Carlo probability was estimated at <0.0001, so that the conditional probability ratio could not be reliably estimated.

### Generalization of model of Wu *et al*. [11]

**Fig 2:**
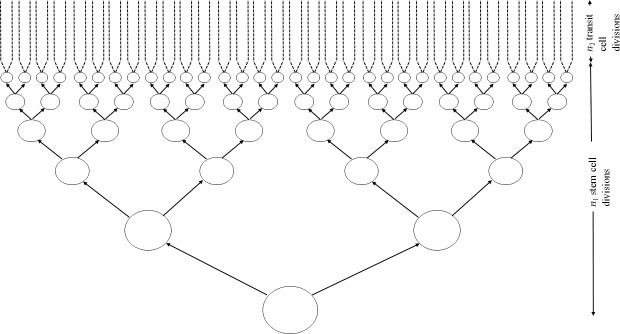
Schematic diagram of stem cell model corresponding to that of Wu *et al*. [11]. The single initial stem cell divides symmetrically *n*_1_ times to produce $2^{n_{1}}$ stem cells. Each cell then divides asymmetrically *n*_2_ times.

As in the above model, this generalized model also assumes a division into stem and transit lineages, and postulates a model in which cancer arises in a specific tissue if there arises a cell with *k* critical driver mutations in particular genes, but only within the stem cell population. As with the model of Wu *et al*. [11], it is assumed that the stem cell divisions are symmetric, each producing two daughter stem cells, and also that there are asymmetric divisions, each producing one daughter stem cell and a daughter non-stem cell; the latter cells are assumed to be irrelevant to the carcinogenic process because of the premises of a limited lifespan and no competition with stem cells for residence in the stem-cell niche. Wu *et al*. [11] assumed that the symmetric stem cell divisions happen first, in this respect contrasting with other models of stem-cell carcinogenesis [6]; however, there is nothing in the mathematical development of Wu *et al*. [11] that makes use of this assumption. We shall not assume that this is necessarily the case either. There are *n*_1_ symmetric stem cell divisions and *n*_2_ asymmetric stem-cell divisions, each stem cell resulting in a total population after the *N* = *n*_1_ + *n*_2_ divisions in the tissue of $T_{s+t}=2^{n_{1}+n_{2}}$ cells, containing a subpopulation of $T_{s}=2^{n_{1}}$ stem cells. In contrast to the model of Wu *et al*. [11], at each stem cell division *i*, whether symmetric or asymmetric, the daughter stem cell is subject to mutation in each critical gene, either from some specific dominant mutagen, the “mutagen-induced” (“extrinsic”) sort, with probability *u*_*M*,*i*_, or resulting from the “spontaneous” (“intrinsic”) sort associated with miscellaneous other endogenous and exogenous mutagenic processes, with probability *u*_*S*,*i*_. This is illustrated schematically in Fig 2. It should be noted that since the number of stem cell divisions, *n*_1_, is generally assumed to be somewhat less than the number of transit cell divisions, *n*_2_ (Table 2), at least in the scheme outlined by Wu *et al*. [11] with stem cell divisions occurring first, over much of the life of the individual the stem cell population is constant.

**Table 2:** Probabilities of cancer for various sites, conditional probability of a cancer being mutagen induced (expression (9)), and the relative risk (expression (10)), assuming a spontaneous mutation rate (*u*_*S*_) = 10^−8^ per cell division, and *k* = 2 to 4 critical cancer genes, mutagen-associated rates increase from 0 after the first third of stem cell divisions, using a generalization of the model of Wu *et al*. [11]. Assumptions as to the number of symmetric (*n*_1_) and asymmetric cell divisions (*n*_2_) are as for the paper of Wu *et al*. [11].

Cancer site	Mutagen-induced mutation rate per cell division (*u*_*M*_) after first third of stem-cell divisions	*k* = 2			*k* = 3			*k* = 4		
		Total probability of cancer	Relative risk	Pr[at least one mutation is mutagen-induced|cancer occurs] (%)	Total probability of cancer	Relative risk	Pr[at least one mutation is mutagen-induced|cancer occurs] (%)	Total probability of cancer	Relative risk	Pr[at least one mutation is mutagen-induced|cancer occurs] (%)
Acute myeloid leukemia	0	1.30 x 10^−2^	1.00	0.0	1.29 x 10^−7^	1.00	0.0	1.27 x 10^−12^	1.00	0.0
	2 x 10^−9^	1.67 x 10^−2^	1.28	22.3	1.88 x 10^−7^	1.46	31.3	2.10 x 10^−12^	1.65	39.4
(*n*_1_ = 27,*n*_2_ = 960)	5 x 10^−9^	2.30 x 10^−2^	1.77	44.1	3.06 x 10^−7^	2.37	57.9	4.03 x 10^−12^	3.17	68.4
	1 x 10^−8^	3.57 x 10^−2^	2.75	64.5	5.99 x 10^−7^	4.64	78.4	9.85 x 10^−12^	7.73	87.1
Basal cell carcinoma	0	1.61 x 10^−1^	1.00	0.0	1.13 x 10^−6^	1.00	0.0	7.21 x 10^−12^	1.00	0.0
	2 x 10^−9^	2.02 x 10^−1^	1.25	24.2	1.64 x 10^−6^	1.46	31.4	1.19 x 10^−11^	1.65	39.5
(*n*_1_ = 32,*n*_2_ = 608)	5 x 10^−9^	2.69 x 10^−1^	1.67	47.7	2.68 x 10^−6^	2.38	57.9	2.28 x 10^−11^	3.17	68.5
	1 x 10^−8^	3.87 x 10^−1^	2.40	69.6	5.23 x 10^−6^	4.65	78.5	5.59 x 10^−11^	7.75	87.1
Colorectal adenocarcinoma	0	6.03 x 10^−1^	1.00	0.0	5.42 x 10^−5^	1.00	0.0	3.18 x 10^−9^	1.00	0.0
	2 x 10^−9^	6.95 x 10^−1^	1.15	33.3	7.90 x 10^−5^	1.46	31.3	5.25 x 10^−9^	1.65	39.4
(*n*_1_ = 28,*n*_2_ = 5840)	5 x 10^−9^	8.07 x 10^−1^	1.34	63.6	1.29 x 10^−4^	2.37	57.8	1.01 x 10^−8^	3.16	68.4
	1 x 10^−8^	9.23 x 10^−1^	1.53	87.4	2.51 x 10^−4^	4.63	78.4	2.46 x 10^−8^	7.72	87.0
Esophageal squamous cell carcinoma	0	2.08 x 10^−4^	1.00	0.0	2.94 x 10^−9^	1.00	0.0	4.14 x 10^−14^	1.00	0.0
	2 x 10^−9^	2.68 x 10^−4^	1.28	22.2	4.28 x 10^−9^	1.46	31.3	6.84 x 10^−14^	1.65	39.4
(*n*_1_ = 20,*n*_2_ = 1390)	5 x 10^−9^	3.71 x 10^−4^	1.78	43.8	6.97 x 10^−9^	2.37	57.8	1.31 x 10^−13^	3.16	68.4
	1 x 10^−8^	5.79 x 10^−4^	2.78	64.0	1.36 x 10^−8^	4.64	78.4	3.20 x 10^−13^	7.73	87.1
Lung adenocarcinoma	0	1.39 x 10^−4^	1.00	0.0	5.01 x 10^−11^	1.00	0.0	1.80 x 10^−17^	1.00	0.0
	2 x 10^−9^	1.80 x 10^−4^	1.30	22.9	7.40 x 10^−11^	1.48	32.3	3.03 x 10^−17^	1.68	40.6
(*n*_1_ = 30,*n*_2_ = 6)	5 x 10^−9^	2.53 x 10^−4^	1.81	44.9	1.22 x 10^−10^	2.45	59.1	5.94 x 10^−17^	3.29	69.6
	1 x 10^−8^	3.99 x 10^−4^	2.87	65.2	2.44 x 10^−10^	4.86	79.4	1.49 x 10^−16^	8.24	87.9
Osteosarcoma	0	3.06 x 10^−7^	1.00	0.0	8.26 x 10^−14^	1.00	0.0	2.23 x 10^−20^	1.00	0.0
	2 x 10^−9^	3.98 x 10^−7^	1.30	23.2	1.23 x 10^−13^	1.48	32.6	3.77 x 10^−20^	1.69	40.9
(*n*_1_ = 22,*n*_2_ = 5)	5 x 10^−9^	5.59 x 10^−7^	1.83	45.3	2.04 x 10^−13^	2.47	59.5	7.44 x 10^−20^	3.34	70.1
	1 x 10^−8^	8.88 x 10^−7^	2.90	65.5	4.08 x 10^−13^	4.95	79.8	1.88 x 10^−19^	8.43	88.1
Thyroid papillary/follicular carcinoma	0	7.31 x 10^−6^	1.00	0.0	2.41 x 10^−12^	1.00	0.0	7.96 x 10^−19^	1.00	0.0
	2 x 10^−9^	9.49 x 10^−6^	1.30	23.0	3.57 x 10^−12^	1.48	32.4	1.34 x 10^−18^	1.69	40.7
(*n*_1_ = 26,*n*_2_ = 7)	5 x 10^−9^	1.33 x 10^−5^	1.82	45.0	5.91 x 10^−12^	2.45	59.2	2.63 x 10^−18^	3.31	69.8
	1 x 10^−8^	2.10 x 10^−5^	2.88	65.3	1.18 x 10^−11^	4.89	79.5	6.60 x 10^−18^	8.29	87.9

The model of Wu *et al*. [11] does not distinguish the two types of mutation, and as is appropriate for a model using only the “intrinsic” mutation rate, does not allow for variations in mutation rate over time (by cell division generation or loss/competition of mutated cells). Each of the mutational events taking place during stem cell division is statistically independent of the others, conditional on the mutations that have already taken place in a particular lineage, and assumed to be irreversible. Let *S*_*g*_ and *M*_*g*_ denote the number of spontaneous and mutagen-induced mutations that have accumulated in a given lineage in generation *g*. Then we have the recurrence relation:

$$\begin{array}{l} P\left(S_{g+1}=s,M_{g+1}=m|{\left(u_{S,i}\right)}_{i=1}^{g+1},{\left(u_{M,i}\right)}_{i=1}^{g+1}\right)\\ =\sum_{j=0}^{s}\sum_{l=0}^{m}\left[\begin{array}{l} \frac{(k-j-l)!}{(s-j)!(m-l)!(k-s-m)!}u_{S,g+1}{}^{s-j}u_{M,g+1}{}^{m-l}{\left(1-u_{S,g+1}-u_{M,g+1}\right)}^{k-s-m}\text{x}\\ P\left(S_{g}=j,M_{g}=l|{\left(u_{S,i}\right)}_{i=1}^{g},{\left(u_{M,i}\right)}_{i=1}^{g}\right) \end{array}\right] \end{array}$$ (5)

This assumes that at each cell division, two daughter cells are produced, whether in the stem cell lineage or a combination of stem and transit cell lineages, with independently produced sets of mutations in the stem cell daughter(s). So that if the stem cell in generation *g* carries *s* spontaneous mutations and *m* mutagen-induced mutations, the numbers of each sort of mutation in the *k* − *s* − *m* remaining non-mutated critical genes is multinomially distributed (∼ *Multinom*(*k* − *s* − *m*,*u*_*S*,*g*+1_,*u*_*M*,*g*+1_,1 – *u*_*S*,*g*+1_ − *u*_*M*,*g*+1_)). We also have that:

$$P\left(S_{g}=s,M_{g}=m|{\left(u_{S,i}\right)}_{i=1}^{0},{\left(u_{M,i}\right)}_{i=1}^{0}\right)=\{\begin{array}{c} 0\;\text{if}\;s\geq 1\;\text{or}\;m\geq 1\\ 1\;\text{if}\;s=m=0 \end{array}$$ (6)

The probability of cancer is then given by:

$$\begin{array}{l} C_{k}\left(n_{1},n_{2}|{\left(u_{S,i}\right)}_{i=1}^{N},{\left(u_{M,i}\right)}_{i=1}^{N}\right)=1-{\left[1-P\left(S_{N}+M_{N}=k|{\left(u_{S,i}\right)}_{i=1}^{N},{\left(u_{M,i}\right)}_{i=1}^{N}\right)\right]}^{T_{s}}\\ =1-{\left[1-\sum_{s=0}^{k}P\left(S_{N}=s,M_{N}=k-s|{\left(u_{S,i}\right)}_{i=1}^{N},{\left(u_{M,i}\right)}_{i=1}^{N}\right)\right]}^{2^{n_{1}}} \end{array}$$ (7)

Also of interest is the probability that cancer develops in which at least one of the critical mutations is mutagen-induced:

$$C_{k}{}^{m}\left(n_{1},n_{2}|{\left(u_{S,i}\right)}_{i=1}^{N},{\left(u_{M,i}\right)}_{i=1}^{N}\right)=1-{\left[1-\sum_{s=0}^{k-1}P\left(S_{N}=s,M_{N}=k-s|{\left(u_{S,i}\right)}_{i=1}^{N},{\left(u_{M,i}\right)}_{i=1}^{N}\right)\right]}^{2^{n_{1}}}$$ (8)

Therefore the conditional probability, given that cancer develops, that it is due to at least one mutation produced by the specified mutagen is:

$${\mathrm{Pr}}_{M}=\frac{C_{k}{}^{m}\left(n_{1},n_{2}|{\left(u_{S,i}\right)}_{i=1}^{N},{\left(u_{M,i}\right)}_{i=1}^{N}\right)}{C_{k}\left(n_{1},n_{2}|{\left(u_{S,i}\right)}_{i=1}^{N},{\left(u_{M,i}\right)}_{i=1}^{N}\right)}=\frac{1-{\left[1-\sum_{s=0}^{k-1}P\left(S_{N}=s,M_{N}=k-s|{\left(u_{S,i}\right)}_{i=1}^{N},{\left(u_{M,i}\right)}_{i=1}^{N}\right)\right]}^{2^{n_{1}}}}{1-{\left[1-\sum_{s=0}^{k}P\left(S_{N}=s,M_{N}=k-s|{\left(u_{S,i}\right)}_{i=1}^{N},{\left(u_{M,i}\right)}_{i=1}^{N}\right)\right]}^{2^{n_{1}}}}$$ (9)

It may also be of interest to calculate the RR due to the specified mutagen, which is given by the quantity:

$$RR_{M}=\frac{C_{k}\left(n_{1},n_{2}|{\left(u_{S,i}\right)}_{i=1}^{N},{\left(u_{M,i}\right)}_{i=1}^{N}\right)}{C_{k}\left(n_{1},n_{2}|{\left(u_{S,i}\right)}_{i=1}^{N},0\right)}=\frac{1-{\left[1-\sum_{s=0}^{k}P\left(S_{N}=s,M_{N}=k-s|{\left(u_{S,i}\right)}_{i=1}^{N},{\left(u_{M,i}\right)}_{i=1}^{N}\right)\right]}^{2^{n_{1}}}}{1-{\left[1-\sum_{s=0}^{k}P\left(S_{N}=s,M_{N}=k-s|{\left(u_{S,i}\right)}_{i=1}^{N},0\right)\right]}^{2^{n_{1}}}}$$ (10)

It should be noted that the model does not accommodate re-hits of the driver mutations that have already occurred, in other words the addition of further mutations in the same critical genes that have already been mutated (whether spontaneously or mutagen-induced), resulting from various types of endogenous and exogenous mutagens. These can happen, but the labelling of the particular stem cell lineage would not alter, and in particular a cell that already has *s* spontaneous and *m* mutagen-induced mutations would be deemed still to have those numbers of mutations if some of these were re-hit by new mutations, whether spontaneous or mutagen-associated. Nevertheless, such re-hits would be expected to be rare occurrences, and arguably of little relevance practically. The model allows for the mutation rates to vary depending on whether the divisions are symmetric or asymmetric, or equivalently whether the divisions take place occur before or after the first *n*_1_ stem cell divisions. However, to the best of our knowledge there are insufficient data to suggest that the mutations rates vary depending on whether cell division is symmetric or asymmetric, so we shall not investigate this possibility further.

We illustrate the effect of assuming a spontaneous mutation rate of *u*_*S*_ = 10^−8^ per cell division, and mutagen associated rates in the range *u*_*M*_ = 0–10^−8^ per cell division in Table 2, for various specific cancer sites, assuming between *k* = 2 to 4 critical cancer mutations [4]. Table A2 in S1 Appendix presents additional calculations assuming a somewhat higher spontaneous mutation rate, *u*_*S*_ = 10^−6^ per cell division, and correspondingly higher mutagen-associated rates, *u*_*M*_ = 0–10^−6^ per cell division. [At least four genetic targets, including *Ras* and *FAP*, have been identified for colon cancer, although not all of them have mutation or sequence loss that are present in every cancer [16], so that this range for *k* appears reasonable to us.] Table A3 in S1 Appendix shows similar calculations, but with the mutagen-associated mutation rate increasing from 0 at birth, rather than after the first third of stem-cell divisions. The mutagen-associated mutation rate is in the range of 0–100% of the spontaneous mutation rate. This range was chosen to yield the range of relative risks (of between 1.2 and 16) commonly observed for many carcinogens, in particular ionising radiation where relative risks tend to be lower [17], or cigarette smoke where the relative risks [18] approach the upper range of 16 yielded by our modelling assumptions (Table 2, Tables A2 and A3 in S1 Appendix). These assumptions also imply that the total mutation rate *u*_*S*_ + *u*_*M*_ = 10^−8^–10^−7^ per cell division, which is similar to values assumed by Wu *et al*. [11], spanning the range 10^−10^–10^−6^ per cell division. We assume a range of numbers of critical mutations required for cancer of *k* = 2 to 4 (Table 2, Tables A2 and A3 in S1 Appendix) or *k* = 1 to 4 (Tables 3 and 4, and Tables A4, A5 in S1 Appendix), similar to the range assumed by Wu *et al*. [11]. Larger values of the critical number of mutations required for cancer (up to *k* = 7) were also evaluated, but these did not suggest any markedly different findings, so are not reported further. All other parameters (*n*_1_,*n*_2_) are the same as assumed by Wu *et al*. [11]. For simplicity we show the calculations for a subset of the more environmentally modifiable cancer types considered by Wu *et al*. [11], in Table 2 and Tables A2 and A3 in S1 Appendix. In Table A1 in S1 Appendix we estimate risks for all cancer sites considered by Wu *et al*. [11], using essentially the cancer site data of Tomasetti and Vogelstein [7].

**Table 3:** Linear regression analysis of probability[at least one mutation is mutagen-induced | cancer occurs] (dependent variable) versus log_10_[cumulative stem cell divisions] (independent variable). The conditional probability is evaluated (via expression (9)) using a generalization of the model of Wu *et al*. [11] using *k* = 1 to 4 critical cancer mutations, a spontaneous mutation rate of *u*_*S*_ = 10^−8^ per cell division, and a mutagen-induced mutation rate, *u*_*M*_ = 2 x 10^−9^, 5 x 10^−9^ or 1 x 10^−8^ per cell division, mutagen-associated rates increase from 0 after the first third of stem cell divisions. The data used in the regression are given in Table A1 in S1 Appendix.

Number of cancer mutations *k*	Spontaneous mutation rate (*u*_*S*_)	Mutagen-induced mutation rate (*u*_*M*_)	*p*-value of trend / *p*-value of trend with outliers removed^a^	R^2^	Pearson correlation coefficient	Spearman correlation coefficient
1	1 x 10^−8^	2 x 10^−9^	<0.0001 / <0.0001	0.6842	0.8272	0.9336
2	1 x 10^−8^	2 x 10^−9^	0.0356 / 0.1690	0.1435	0.3788	0.0190
3	1 x 10^−8^	2 x 10^−9^	<0.0001 / <0.0001	0.6633	-0.8144	-0.7571
4	1 x 10^−8^	2 x 10^−9^	<0.0001 / <0.0001	0.6636	-0.8146	-0.7543
1	1 x 10^−8^	5 x 10^−9^	<0.0001 / <0.0001	0.6604	0.8127	0.9336
2	1 x 10^−8^	5 x 10^−9^	0.0264 / 0.5146	0.1588	0.3985	0.0243
3	1 x 10^−8^	5 x 10^−9^	<0.0001 / <0.0001	0.6633	-0.8144	-0.7571
4	1 x 10^−8^	5 x 10^−9^	<0.0001 / <0.0001	0.6638	-0.8148	-0.7571
1	1 x 10^−8^	1 x 10^−8^	<0.0001 / <0.0001	0.6395	0.7997	0.8883
2	1 x 10^−8^	1 x 10^−8^	0.0180 / 0.8103	0.1781	0.4220	0.0352
3	1 x 10^−8^	1 x 10^−8^	<0.0001 / <0.0001	0.6633	-0.8144	-0.7571
4	1 x 10^−8^	1 x 10^−8^	<0.0001 / <0.0001	0.6640	-0.8148	-0.7571

^a^*p*-value of trend with all high-leverage datapoints, with Cook’s-distance > 4/[*n*–*p*—1] (*n* = number of datapoints, *p* = number of fitted parameters), removed.

**Table 4:** Linear regression analysis of probability[at least one mutation is mutagen-induced | cancer occurs] (dependent variable) versus smoking-associated cancer risk (using data taken from Doll *et al*. [18]) (independent variable). The conditional probability is evaluated (via expression (9)) using the model of Wu *et al*. [11] using *k* = 1 to 4 critical cancer mutations, a spontaneous mutation rate of *u*_*S*_ = 10^−8^ per cell division, and a mutagen-induced mutation rate, *u*_*M*_ = 2 x 10^−9^, 5 x 10^−9^ or 1 x 10^−8^ per cell division, mutagen-associated rates increase from 0 after the first third of stem cell divisions. The data used in the regression are given in Table A1 in S1 Appendix and in Table 2 of Little *et al*. [9].

Number of cancer mutations *k*	Spontaneous mutation rate (*u*_*S*_)	Mutagen-induced mutation rate (*u*_*M*_)	*p*-value of trend / *p*-value of trend with outliers removed^a^	R^2^	Pearson correlation coefficient	Spearman correlation coefficient
1	1 x 10^−8^	2 x 10^−9^	0.7504 / 0.0460	0.0087	0.3673	0.3416
2	1 x 10^−8^	2 x 10^−9^	0.7405 / 0.3114	0.0095	-0.0884	-0.1993
3	1 x 10^−8^	2 x 10^−9^	0.0768 / 0.3809	0.2380	-0.3631	-0.2153
4	1 x 10^−8^	2 x 10^−9^	0.0768 / 0.3822	0.2380	-0.3631	-0.2339
1	1 x 10^−8^	5 x 10^−9^	0.7690 / 0.0205	0.0075	0.3776	0.3416
2	1 x 10^−8^	5 x 10^−9^	0.7157 / 0.3691	0.0115	-0.0765	-0.1856
3	1 x 10^−8^	5 x 10^−9^	0.0769 / 0.3823	0.2378	-0.3624	-0.2153
4	1 x 10^−8^	5 x 10^−9^	0.0771 / 0.3841	0.2375	-0.3619	-0.2153
1	1 x 10^−8^	1 x 10^−8^	0.7695 / 0.0189	0.0074	0.3673	0.2500
2	1 x 10^−8^	1 x 10^−8^	0.6852 / 0.4387	0.0142	-0.0602	-0.1869
3	1 x 10^−8^	1 x 10^−8^	0.0773 / 0.3836	0.2373	-0.3614	-0.2153
4	1 x 10^−8^	1 x 10^−8^	0.0775 / 0.3852	0.2370	-0.3605	-0.2153

^a^*p*-value of trend with all high-leverage datapoints, with Cook’s-distance > 4/[*n*–*p*—1] (*n* = number of datapoints, *p* = number of fitted parameters), removed.

### Special case of fully-stochastic multistage carcinogenesis model of Little *et al*. [13]

**Fig 3:**
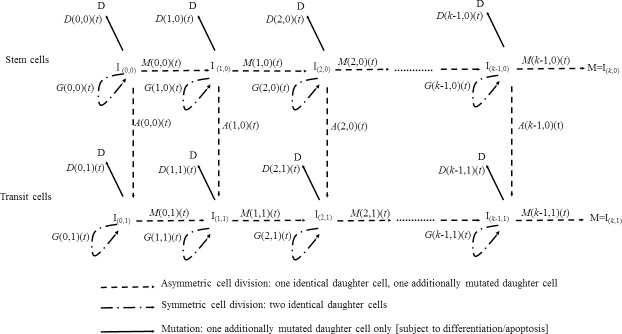
Schematic diagram of generalized cancer model with *k* mutations, allowing for mutations in stem cell and transit cell compartments. This is a special case of the fully-stochastic destabilization model developed by Little *et al*. [13].

We also assessed predictions of a variety of multistage carcinogenesis models, in order to determine the likely effect of intermediate (partially transformed) cell proliferation and death/differentiation, also variations made by assuming that excess mutations affect only the stem cell or transition cell compartment. Stem cells or transit cells can acquire up to *k* successive mutations, at which point they are assumed to become malignant. The model is illustrated schematically in Fig 3. Cells at different stages of the process are labelled by *I*_(*α*,*β*)_, where the first subscript, *α*, represents the number of cancer mutations that the cell has accumulated, the second subscript, *β*, represents whether the cell is a stem cell (*β* = 0) or a transit cell (*β* = 1). At all stages stem or transit cells are allowed to divide symmetrically or differentiate (or undergo apoptosis) at rates *G*(*α*,*β*) and *D*(*α*,*β*), respectively. Each stem or transit cell can asymmetrically divide into an equivalent daughter cell and another cell with an extra cancer mutation at rate *M*(*α*,*β*). Likewise, stem cells can also asymmetrically divide into an equivalent daughter cell and a transit cell at rate *A*(*α*,0). The model assumes that at age 0 there is a single stem cell, and no transit cells. This model is a special case of models developed by Little *et al*. [13] and used to fit to population retinoblastoma data. This differs from the otherwise very similar models of Little *et al*. [19] and Little and Wright [20] only in that the previous models assumed a deterministic (non-stochastic) untransformed stem cell population. In Fig 3 stem cells correspond to the upper horizontal axis, whereas transit cells are given by the lower horizontal axis. The acquisition of carcinogenic mutations amounts to moving horizontally (left to right) via successive symmetric division processes both for stem (upper axis) and transit cells (lower axis) in Fig 3, whereas the asymmetric division that produces for each stem cell a single daughter stem and transit cell, and which can happen in principle to a stem cell with any number of accumulated mutations, amounts to moving vertically (top to bottom) in this figure. Further details on the mathematical assumptions and the numerical solution of the governing partial differential equations are given in Little *et al*. [13]. We shall assume that during gestation (assumed to be of length *L*_*g*_ = 0.728 years [38 weeks]) the stem cell population divides at a rate:

$$G(0,0)=\ln[2^{n_{1}}]/L_{g}$$ (11)

per cell per year, with cell differentiation/apoptosis rate *D*(0,0) = 0, so that at the end of gestation the expected number of stem cells $\approx 2^{n_{1}}$. The lifetime of the individual is assumed to be of duration *L*_*t*_ = 80 years. We assume that the transit cell population has a slightly faster growth rate:

$$G(0,1)=1+G(0,0)=1+\ln[2^{n_{1}}]/L_{g}$$ (12)

and again the cell differentiation/apoptosis rate is 0. After gestation we generally assume that all *G*(*α*,*β*) and *D*(*α*,*β*) are 0; in particular this implies that after birth the stem-cell and transit-cell populations are approximately constant, although both are random processes, so that there will be modest fluctuations in the size of each cell population. However, to allow for the possibility of intermediate (partially transformed) stem and transit cells being subject to competing processes of proliferation and differentiation/apoptosis, a process that is incorporated in many recently developed mathematical cancer models [13, 19–23], we conduct sensitivity analysis in Table 5 in which we assume a birth/death process for all intermediate cell compartments, with *G*(*α*,*β*) = 1.1 /cell / year and *D*(*α*,*β*) = 0.71 /cell / year for (*α*,*β*) ∉ {(0,0),(0,1)}; these values are derived from analysis of lung cancer mortality data [24]. Assuming that the transit cell population at the end of gestation is $\approx T_{g}=p2^{n_{1}}$, implies that the stem cell→transit cell transition rate must be:

$$A(0,0)=T_{g}(G(0,0)-G(0,1))/(\exp[G(0,0)L_{g}]-\exp[G(0,1)L_{g}])$$ (13)

**Table 5:** Probability of cancer and relative risk using generalized multistage model, allowing for mutations in stem cell and transit cell compartments. This is a special case of the fully-stochastic model developed by Little *et al* [13].

	Stem cell mutation rate	Transit cell mutation rate	Total probability of cancer	Relative risk	Total probability of cancer	Relative risk	Total probability of cancer	Relative risk	Total probability of cancer	Relative risk	Total probability of cancer	Relative risk	Total probability of cancer	Relative risk
			*k* = 2		*k* = 3		*k* = 4		*k* = 2		*k* = 3		*k* = 4	
			Standard model [no intermediate cell growth, death/differentiation]						Model with intermediate compartment cell growth 1.1 /cell /y, cell death 0.71 /cell /y					
Acute myeloid leukemia (*n*_1_ = 27, *n*_2_ = 960)	1.00 x 10^−8^	1.00 x 10^−6^	9.41 x 10^−1^	1	3.91 x 10^−3^	1	7.13 x 10^−7^	1	1.00	1	1.00	1	9.99 x 10^−1^	1
	1.20 x 10^−8^	1.20 x 10^−6^	9.56 x 10^−1^	1.02	6.17 x 10^−3^	1.58	1.30 x 10^−6^	1.82	1.00	1.00	1.00	1.00	9.99 x 10^−1^	1.00
	1.50 x 10^−8^	1.50 x 10^−6^	9.70 x 10^−1^	1.03	1.10 x 10^−2^	2.80	2.95 x 10^−6^	4.14	1.00	1.00	1.00	1.00	9.99 x 10^−1^	1.00
	2.00 x 10^−8^	2.00 x 10^−6^	9.82 x 10^−1^	1.04	2.35 x 10^−2^	6.00	8.08 x 10^−6^	11.3	1.00	1.00	1.00	1.00	9.99 x 10^−1^	1.00
Basal cell carcinoma (*n*_1_ = 32, *n*_2_ = 608)	1.00 x 10^−8^	1.00 x 10^−6^	9.95 x 10^−1^	1	3.09 x 10^−2^	1	1.75 x 10^−6^	1	1.00	1	1.00	1	1.00	1
	1.20 x 10^−8^	1.20 x 10^−6^	9.96 x 10^−1^	1.00	4.80 x 10^−2^	1.55	4.10 x 10^−6^	2.35	1.00	1.00	1.00	1.00	1.00	1.00
	1.50 x 10^−8^	1.50 x 10^−6^	9.98 x 10^−1^	1.00	8.26 x 10^−2^	2.67	1.44 x 10^−5^	8.17	1.00	1.00	1.00	1.00	1.00	1.00
	2.00 x 10^−8^	2.00 x 10^−6^	9.99 x 10^−1^	1.00	1.63 x 10^−1^	5.28	3.76 x 10^−5^	21.5	1.00	1.00	1.00	1.00	1.00	1.00
Colorectal adenocarcinoma (*n*_1_ = 28, *n*_2_ = 5840)	1.00 x 10^−8^	1.00 x 10^−6^	9.99 x 10^−1^	1	6.37 x 10^−1^	1	2.10 x 10^−3^	1	1.00	1	1.00	1	1.00	1
	1.20 x 10^−8^	1.20 x 10^−6^	9.99 x 10^−1^	1.00	7.35 x 10^−1^	1.15	3.78 x 10^−3^	1.81	1.00	1.00	1.00	1.00	1.00	1.00
	1.50 x 10^−8^	1.50 x 10^−6^	1.00	1.00	8.32 x 10^−1^	1.31	8.04 x 10^−3^	3.85	1.00	1.00	1.00	1.00	1.00	1.00
	2.00 x 10^−8^	2.00 x 10^−6^	1.00	1.00	9.15 x 10^−1^	1.44	2.19 x 10^−2^	10.5	1.00	1.00	1.00	1.00	1.00	1.00
Esophageal squamous cell carcinoma (*n*_1_ = 20, *n*_2_ = 1390)	1.00 x 10^−8^	1.00 x 10^−6^	2.09 x 10^−1^	1	9.32 x 10^−5^	1	2.55 x 10^−8^	1	9.90 x 10^−1^	1	9.78 x 10^−1^	1	9.18 x 10^−1^	1
	1.20 x 10^−8^	1.20 x 10^−6^	2.65 x 10^−1^	1.27	1.47 x 10^−4^	1.58	4.86 x 10^−8^	1.91	9.92 x 10^−1^	1.00	9.81 x 10^−1^	1.00	9.27 x 10^−1^	1.01
	1.50 x 10^−8^	1.50 x 10^−6^	3.49 x 10^−1^	1.67	2.63 x 10^−4^	2.82	1.02 x 10^−7^	3.99	9.93 x 10^−1^	1.00	9.84 x 10^−1^	1.01	9.37 x 10^−1^	1.02
	2.00 x 10^−8^	2.00 x 10^−6^	4.75 x 10^−1^	2.28	5.70 x 10^−4^	6.12	2.81 x 10^−7^	11.0	9.95 x 10^−1^	1.00	9.88 x 10^−1^	1.01	9.48 x 10^−1^	1.03
Lung carcinoma (*n*_1_ = 30, *n*_2_ = 6)	1.00 x 10^−8^	1.00 x 10^−6^	5.03 x 10^−3^	1	8.80 x 10^−20^	1	4.45 x 10^−25^	1	9.96 x 10^−1^	1	9.53 x 10^−1^	1	4.02 x 10^−4^	1
	1.20 x 10^−8^	1.20 x 10^−6^	6.87 x 10^−3^	1.37	1.28 x 10^−19^	1.46	5.73 x 10^−25^	1.29	9.97 x 10^−1^	1.00	9.59 x 10^−1^	1.01	6.20 x 10^−4^	1.54
	1.50 x 10^−8^	1.50 x 10^−6^	1.02 x 10^−2^	2.02	2.06 x 10^−19^	2.34	7.95 x 10^−25^	1.79	9.97 x 10^−1^	1.00	9.65 x 10^−1^	1.01	1.21 x 10^−3^	3.01
	2.00 x 10^−8^	2.00 x 10^−6^	1.71 x 10^−2^	3.40	3.88 x 10^−19^	4.41	1.25 x 10^−24^	2.80	9.98 x 10^−1^	1.00	9.70 x 10^−1^	1.02	2.67 x 10^−3^	6.65
Osteosarcoma (*n*_1_ = 22, *n*_2_ = 5)	1.00 x 10^−8^	1.00 x 10^−6^	1.37 x 10^−5^	1	5.21 x 10^−20^	1	6.45 x 10^−22^	1	4.64 x 10^−1^	1	5.67 x 10^−2^	1	7.51 x 10^−7^	1
	1.20 x 10^−8^	1.20 x 10^−6^	1.88 x 10^−5^	1.37	7.62 x 10^−20^	1.46	8.31 x 10^−22^	1.29	4.98 x 10^−1^	1.07	6.46 x 10^−2^	1.14	1.16 x 10^−6^	1.55
	1.50 x 10^−8^	1.50 x 10^−6^	2.79 x 10^−5^	2.03	1.22 x 10^−19^	2.35	1.15 x 10^−21^	1.79	5.43 x 10^−1^	1.17	7.51 x 10^−2^	1.32	2.13 x 10^−6^	2.84
	2.00 x 10^−8^	2.00 x 10^−6^	4.71 x 10^−5^	3.44	2.29 x 10^−19^	4.38	1.81 x 10^−21^	2.80	6.05 x 10^−1^	1.30	8.96 x 10^−2^	1.58	5.05 x 10^−6^	6.72
Thyroid papillary/ follicular carcinoma (*n*_1_ = 26, *n*_2_ = 7)	1.00 x 10^−8^	1.00 x 10^−6^	4.30 x 10^−4^	1	1.38 x 10^−19^	1	1.72 x 10^−23^	1	9.52 x 10^−1^	1	6.29 x 10^−1^	1	4.64 x 10^−5^	1
	1.20 x 10^−8^	1.20 x 10^−6^	5.88 x 10^−4^	1.37	2.01 x 10^−19^	1.45	2.21 x 10^−23^	1.29	9.58 x 10^−1^	1.01	6.58 x 10^−1^	1.05	7.47 x 10^−5^	1.61
	1.50 x 10^−8^	1.50 x 10^−6^	8.74 x 10^−4^	2.03	3.24 x 10^−19^	2.34	3.07 x 10^−23^	1.79	9.65 x 10^−1^	1.01	6.90 x 10^−1^	1.10	1.38 x 10^−4^	2.98
	2.00 x 10^−8^	2.00 x 10^−6^	1.48 x 10^−3^	3.43	6.05 x 10^−19^	4.37	4.81 x 10^−23^	2.80	9.73 x 10^−1^	1.02	7.26 x 10^−1^	1.16	3.00 x 10^−4^	6.46
			Model with mutagen-associated stem cell mutations only [no intermediate cell growth, death/differentiation]						Model with mutagen-associated transit cell mutations only [no intermediate cell growth, death/differentiation]					
Acute myeloid leukemia (*n*_1_ = 27, *n*_2_ = 960)	1.00 x 10^−8^	1.00 x 10^−6^	9.41 x 10^−1^	1	3.91 x 10^−3^	1	1.07 x 10^−8^	1	9.41 x 10^−1^	1	3.91 x 10^−3^	1	7.13 x 10^−7^	1
	1.20 x 10^−8^ ^a^	1.20 x 10^−6^ ^b^	9.41 x 10^−1^	1.00	3.91 x 10^−3^	1.00	1.07 x 10^−8^	1.00	9.56 x 10^−1^	1.02	6.17 x 10^−3^	1.58	1.30 x 10^−6^	1.82
	1.50 x 10^−8^ ^a^	1.50 x 10^−6^ ^b^	9.41 x 10^−1^	1.00	3.92 x 10^−3^	1.00	1.07 x 10^−8^	1.00	9.70 x 10^−1^	1.03	1.10 x 10^−2^	2.80	2.95 x 10^−6^	4.14
	2.00 x 10^−8^ ^a^	2.00 x 10^−6^ ^b^	9.41 x 10^−1^	1.00	3.92 x 10^−3^	1.00	1.07 x 10^−8^	1.00	9.82 x 10^−1^	1.04	2.35 x 10^−2^	6.00	8.08 x 10^−6^	11.3
Basal cell carcinoma (*n*_1_ = 32, *n*_2_ = 608)	1.00 x 10^−8^	1.00 x 10^−6^	9.95 x 10^−1^	1	3.09 x 10^−2^	1	9.04 x 10^−18^	1	9.95 x 10^−1^	1	3.09 x 10^−2^	1	1.75 x 10^−6^	1
	1.20 x 10^−8^ ^a^	1.20 x 10^−6^ ^b^	9.95 x 10^−1^	1.00	3.09 x 10^−2^	1.00	9.29 x 10^−18^	1.03	9.96 x 10^−1^	1.00	4.80 x 10^−2^	1.55	4.10 x 10^−6^	2.35
	1.50 x 10^−8^ ^a^	1.50 x 10^−6^ ^b^	9.95 x 10^−1^	1.00	3.10 x 10^−2^	1.00	9.67 x 10^−18^	1.07	9.98 x 10^−1^	1.00	8.26 x 10^−2^	2.67	1.44 x 10^−5^	8.22
	2.00 x 10^−8^ ^a^	2.00 x 10^−6^ ^b^	9.95 x 10^−1^	1.00	3.10 x 10^−2^	1.00	1.03 x 10^−17^	1.14	9.99 x 10^−1^	1.00	1.63 x 10^−1^	5.28	3.72 x 10^−5^	21.3
Colorectal adenocarcinoma (*n*_1_ = 28, *n*_2_ = 5840)	1.00 x 10^−8^	1.00 x 10^−6^	9.99 x 10^−1^	1	6.37 x 10^−1^	1	2.10 x 10^−3^	1	9.99 x 10^−1^	1	6.37 x 10^−1^	1	2.09 x 10^−3^	1
	1.20 x 10^−8^ ^a^	1.20 x 10^−6^ ^b^	9.99 x 10^−1^	1.00	6.37 x 10^−1^	1.00	2.10 x 10^−3^	1.00	9.99 x 10^−1^	1.00	7.35 x 10^−1^	1.15	3.78 x 10^−3^	1.81
	1.50 x 10^−8^ ^a^	1.50 x 10^−6^ ^b^	9.99 x 10^−1^	1.00	6.37 x 10^−1^	1.00	2.10 x 10^−3^	1.00	1.00	1.00	8.32 x 10^−1^	1.31	8.03 x 10^−3^	3.85
	2.00 x 10^−8^ ^a^	2.00 x 10^−6^ ^b^	9.99 x 10^−1^	1.00	6.37 x 10^−1^	1.00	2.10 x 10^−3^	1.00	1.00	1.00	9.15 x 10^−1^	1.44	2.19 x 10^−2^	10.5
Esophageal squamous cell carcinoma (*n*_1_ = 20, *n*_2_ = 1390)	1.00 x 10^−8^	1.00 x 10^−6^	2.09 x 10^−1^	1	9.32 x 10^−5^	1	1.56 x 10^−9^	1	2.09 x 10^−1^	1	9.32 x 10^−5^	1	2.55 x 10^−8^	1
	1.20 x 10^−8^ ^a^	1.20 x 10^−6^ ^b^	2.09 x 10^−1^	1.00	9.32 x 10^−5^	1.00	1.56 x 10^−9^	1.00	2.65 x 10^−1^	1.27	1.47 x 10^−4^	1.58	4.86 x 10^−8^	1.91
	1.50 x 10^−8^ ^a^	1.50 x 10^−6^ ^b^	2.09 x 10^−1^	1.00	9.33 x 10^−5^	1.00	1.56 x 10^−9^	1.00	3.49 x 10^−1^	1.67	2.63 x 10^−4^	2.82	1.01 x 10^−7^	3.98
	2.00 x 10^−8^ ^a^	2.00 x 10^−6^ ^b^	2.09 x 10^−1^	1.00	9.34 x 10^−5^	1.00	1.56 x 10^−9^	1.00	4.75 x 10^−1^	2.27	5.70 x 10^−4^	6.11	2.81 x 10^−7^	11.0
Lung carcinoma (*n*_1_ = 30, *n*_2_ = 6)	1.00 x 10^−8^	1.00 x 10^−6^	5.03 x 10^−3^	1	8.80 x 10^−20^	1	4.45 x 10^−25^	1	5.03 x 10^−3^	1	8.80 x 10^−20^	1	4.45 x 10^−25^	1
	1.20 x 10^−8^ ^a^	1.20 x 10^−6^ ^b^	5.03 x 10^−3^	1.00	9.09 x 10^−20^	1.03	4.45 x 10^−25^	1.00	6.87 x 10^−3^	1.37	1.24 x 10^−19^	1.41	5.73 x 10^−25^	1.29
	1.50 x 10^−8^ ^a^	1.50 x 10^−6^ ^b^	5.04 x 10^−3^	1.00	9.53 x 10^−20^	1.08	4.45 x 10^−25^	1.00	1.02 x 10^−2^	2.02	1.90 x 10^−19^	2.16	7.95 x 10^−25^	1.79
	2.00 x 10^−8^ ^a^	2.00 x 10^−6^ ^b^	5.05 x 10^−3^	1.00	1.03 x 10^−19^	1.17	4.45 x 10^−25^	1.00	1.70 x 10^−2^	3.39	3.31 x 10^−19^	3.76	1.25 x 10^−24^	2.80
Osteosarcoma (*n*_1_ = 22, *n*_2_ = 5)	1.00 x 10^−8^	1.00 x 10^−6^	1.37 x 10^−5^	1	5.21 x 10^−20^	1	6.45 x 10^−22^	1	1.37 x 10^−5^	1	5.21 x 10^−20^	1	6.45 x 10^−22^	1
	1.20 x 10^−8^ ^a^	1.20 x 10^−6^ ^b^	1.37 x 10^−5^	1.00	5.39 x 10^−20^	1.03	6.45 x 10^−22^	1.00	1.88 x 10^−5^	1.37	7.37 x 10^−20^	1.41	8.31 x 10^−22^	1.29
	1.50 x 10^−8^ ^a^	1.50 x 10^−6^ ^b^	1.37 x 10^−5^	1.00	5.64 x 10^−20^	1.08	6.45 x 10^−22^	1.00	2.78 x 10^−5^	2.03	1.13 x 10^−19^	2.17	1.15 x 10^−21^	1.79
	2.00 x 10^−8^ ^a^	2.00 x 10^−6^ ^b^	1.38 x 10^−5^	1.00	6.07 x 10^−20^	1.16	6.45 x 10^−22^	1.00	4.70 x 10^−5^	3.43	1.95 x 10^−19^	3.75	1.81 x 10^−21^	2.80
Thyroid papillary/follicular carcinoma (*n*_1_ = 26, *n*_2_ = 7)	1.00 x 10^−8^	1.00 x 10^−6^	4.30 x 10^−4^	1	1.38 x 10^−19^	1	1.72 x 10^−23^	1	4.30 x 10^−4^	1	1.38 x 10^−19^	1	1.72 x 10^−23^	1
	1.20 x 10^−8^ ^a^	1.20 x 10^−6^ ^b^	4.30 x 10^−4^	1.00	1.43 x 10^−19^	1.03	1.72 x 10^−23^	1.00	5.88 x 10^−4^	1.37	1.94 x 10^−19^	1.40	2.21 x 10^−23^	1.29
	1.50 x 10^−8^ ^a^	1.50 x 10^−6^ ^b^	4.30 x 10^−4^	1.00	1.50 x 10^−19^	1.08	1.72 x 10^−23^	1.00	8.73 x 10^−4^	2.03	2.98 x 10^−19^	2.15	3.07 x 10^−23^	1.79
	2.00 x 10^−8^ ^a^	2.00 x 10^−6^ ^b^	4.31 x 10^−4^	1.00	1.62 x 10^−19^	1.17	1.72 x 10^−23^	1.00	1.47 x 10^−3^	3.43	5.13 x 10^−19^	3.71	4.81 x 10^−23^	2.80

^a^in model assuming mutation-associated transit cell mutations only, these take the value 1.00 x 10^−8^

^b^in model assuming mutation-associated stem cell mutations only, these take the value 1.00 x 10^−6^.

We assume a ratio of transit:stem cells at the end of gestation of 1:100, i.e., *p* = 0.01, throughout. In order to derive the mutation rates *M*(*α*,*β*), we note that the probability of a mutation per asymmetric cell division, whether in stem or transit cells, over the expected duration, *L*_*t*_ / *n*_2_, between asymmetric cell divisions, is *u* = 1 − exp[−*M*(*α*,*β*)*L*_*t*_ / *n*_2_]. Therefore:

$$M(\alpha ,\beta )=-n_{2}\ln[1-u]/L_{t}$$ (14)

Frank *et al*. discuss the possibility that “transit cells [in the colon] may require mutations to avoid sloughing to cause cancer. For example, an additional mutation that makes a transit cell surface sticky may prevent it from shedding” [6]; however, Frank *et al*. [6] did not actually fit such a model. We conduct additional sensitivity analysis in Table A6 in S1 Appendix in which we assess the implications of a model in which there is an additional mutational stage required for transit cells than for stem cells.

Transmissible genomic instability, for which there is experimental evidence after radiation exposure [25, 26], implies that certain sorts of mutation can result in a long-lasting increase in mutation rate. The role of genomic instability is particularly well established for colon cancer; chromosomal instability is present in about 85% of non-familial colon cancers, and microsatellite instability is associated with most of the remaining carcinomas [27–29]. We therefore assess the effect of non-homogenous mutation rates on the relative risk of colon adenocarcinoma, whereby the ratio between successive mutation rates, whether for the stem cells or transit cells is given by *d* = *M*(*α* + 1,*β*) / *M*(*α*,*β*), so that:

$$M(\alpha ,\beta )=d^{\alpha }M(0,\beta )$$ (15)

**Fig 4:**
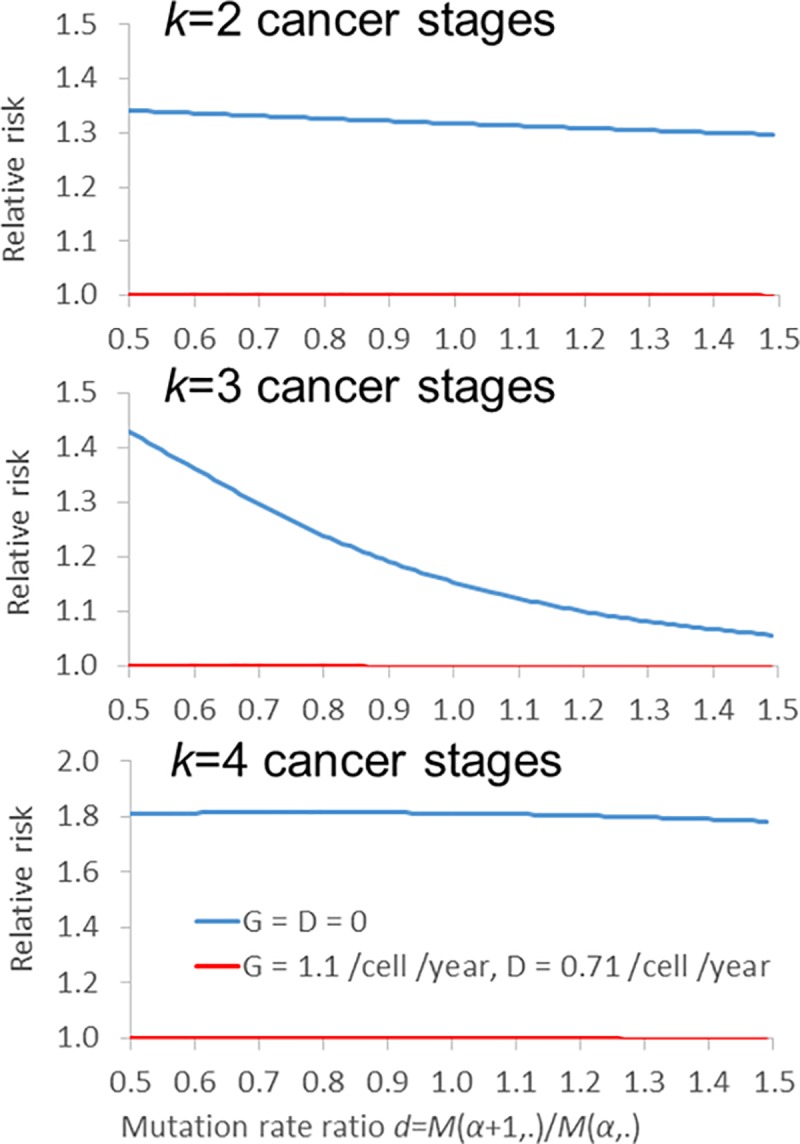
Relative risk for colorectal adenocarcinoma using generalized multistage model allowing for genomic destabilization/stabilization, via progressive increase or decrease in mutation rates (via parameter *d* in expression (15)). The relative risk is evaluated using *k* = 2, *k* = 3 or *k* = 4 cancer stages, a spontaneous mutation rate of 10^−8^ per cell division (for *k* = 2 a spontaneous mutation rate of 10^−10^ per cell division), and a mutagen-induced mutation rate of 2 x 10^−9^ per cell division (for *k* = 2 a mutagen-induced mutation rate of 2 x 10^−11^ per cell division), and mutagen-associated rates increase from 0 after the first third of life (~26 years). The data used for colorectal adenocarcinoma are as given in Table A1 in S1 Appendix. This is a special case of the fully-stochastic model developed by Little *et al* [13].

The case in which *d* > 1 implies that with each acquired mutation the mutation rate (per cell per unit time) increases, so that after the first mutation the second mutation is acquired somewhat faster (per cell per unit time), and after the second mutation, the third mutations is acquired even faster (per cell per unit time), somewhat analogous to transmissible genomic instability, whereas *d* < 1 implies that with each acquired mutation the mutation rate (per cell per unit time) decreases, the opposite of this process. We plot the relative risks implied by such a process in Fig 4, varying *d* over the range from 0.5 to 1.5 with values of the number of critical mutations, *k*, between 2 and 4; a lower mutation rate, both for stem and transit cells, is used for *k* = 2 than for *k* = 3,4 in order to avoid the probability of cancer saturating (at 1).

A test can be made of the assumption that Pr_*M*_, as given by expression (1) (for the generalization of the model of Frank *et al*. [6]) and by expression (9) (for the generalization of the model of Wu *et al*. [11]), may be correlated with the susceptibility of a tissue to environmental mutagenic factors, using radiation-associated and smoking-associated cancer risk as examples of such factors, both being mutagens that induce a large number of types of cancer [17, 18]. We consider radiation-exposure induced solid cancer incidence risk (REIC) at 1 Gy evaluated by the United Nations Scientific Committee on the Effects of Atomic Radiation (UNSCEAR) [Table 70 in Annex A of [17]] for various cancer sites, and as also quoted by Little *et al*. [Table 1 in [9]]; for leukemia we use radiation-exposure induced cancer death risk (REID) evaluated by UNSCEAR [Table 65 in Annex A of [17]]; mortality risk is used because leukemia incidence was not evaluated in the latest Japanese atomic bomb survivor Life Span Study (LSS) cancer incidence report [30], a preliminary version of which formed the basis of the UNSCEAR evaluations [17]. We use both the International Commission on Radiological Protection (ICRP) recommended [31] cancer-site specific weighting of excess absolute risk (EAR) *vs* excess relative risk (ERR) models (Table A4 in S1 Appendix), and the Biological Effects of Ionizing Radiation (BEIR VII) committee recommended [32] cancer-site specific weighting of EAR vs ERR models are employed (Table A5 in S1 Appendix). While these risks may be taken as representing those associated with exposure at high doses and high dose rates, they are likely to be proportional to risks associated with environmental and occupational levels of radiation exposure [31, 33, 34]. Proceeding along the lines of analysis conducted by Little *et al*. [9] we tested whether the conditional probability, Pr_*M*_, of cancer being mutagen-induced, as given by expression (9), was associated with estimates of the cumulative number of stem-cell divisions in that tissue. In order to do this we regress the conditional probability Pr_*M*_, in relation to the log of the cumulative number of cell divisions, ln[*D*], given by:

$${\mathrm{Pr}}_{M}=\alpha_{0}+\alpha_{1}\ln[D]+\varepsilon$$ (16)

For REIC, we fit a model in which:

$$REIC=\alpha_{0}+\alpha_{1}{\mathrm{Pr}}_{M}+\varepsilon$$ (17)

Likewise, we assess the correlations of smoking-associated cancer risk using data on differences in mortality rates between current and former smokers, *Sm*_*diff*_, in the British doctors’ cohort [18], as also shown in Table 2 of Little *et al*. [9], by fitting a model in which:

$$Sm_{diff}=\alpha_{0}+\alpha_{1}{\mathrm{Pr}}_{M}+\varepsilon$$ (18)

Tables 3 and 4 and Tables A4 and A5 in S1 Appendix record the results of these regression analyses, based on the data referred to above and also in Table A1 in S1 Appendix. We are mainly interested in the significance of the regression coefficient *α*_1_ in expressions (16)-(18). Linear regressions are performed via ordinary least squares [35], using R [36]. The *p*-values shown in Tables 3 and 4 and Tables A4 and A5 in S1 Appendix are estimated using an *F*-test [35], and are in relation to the trend parameter (*α*_1_). We also estimate Pearson and Spearman correlation coefficients between Pr_*M*_ and these other variables, ln[*D*], *REIC*, and *Sm*_*diff*_. We assess the influence of high-leverage datapoints by assessing the difference made by removing those points from each regression with Cook’s distance [37] exceeding 4 / [*n* − *p* − 1], where *n* = number of relevant datapoints, *p* = number of fitted parameters, a generally used threshold [38].

## Results

Table 1 demonstrates that if the generalization of the model of Frank *et al*. is employed, there is a substantial variation in cancer risks, by at least 4 orders of magnitude, depending on the assumed number of critical mutations required for cancer, *k*, and the stem-cell and transition-cell mutation rates. If only a single cancer mutation, *k* = 1, is assumed the total probability of cancer is between 88–100%, irrespective of the assumed mutation rates, but the cancer probability is lower, generally less than 80%, if *k* = 2 or *k* = 3 cancer mutations. If *k* = 3 and stem cells have 100-fold lower mutation rates than transit cells (upper part of Table 1), the cancer probability is less than 0.6%. However, in most cases the conditional probabilities, Pr_*M*_, as given by expression (1), of cancer being mutagen-induced, are within a factor of about 10, ranging between 7–81%. The only situations in which this is not the case are when a single mutation is required for cancer and the baseline mutation rates are sufficiently high that cancer develops during the first third of cell divisions, before any external mutagen is assumed to be present. The relative risks, *RR*_*M*_, as given by expression (4), are also moderately stable, spanning the range 1.0–4.5 (Table 1). In general, conditional on cancer developing, the probability is low (<0.5%) of all the relevant mutations being derived from stem cell-associated mutations (Table 1). However, particularly for cancers with 2 or 3 critical mutations, a substantial proportion of cancers, in some cases 100%, have at least one mutation derived from a mutated stem cell.

If now the generalization of the model of Wu *et al*. is employed, the results of Table 2, Tables A2 and A3 in S1 Appendix again demonstrate that cancer risks vary quite substantially, by about 12 orders of magnitude, depending on the assumed number of critical mutations required for cancer, *k*, and the spontaneous and mutagen-associated mutation rates. However, again the conditional probabilities, Pr_*M*_, as given by expression (9), are reasonably stable, ranging between 22–98% (Table 2, Tables A2 and A3 in S1 Appendix). Relative risks, *RR*_*M*_, as given by expression (10), are also fairly consistent, spanning the range 1.2–16.0 (Table 2, Tables A2 and A3 in S1 Appendix).

**Fig 5:**
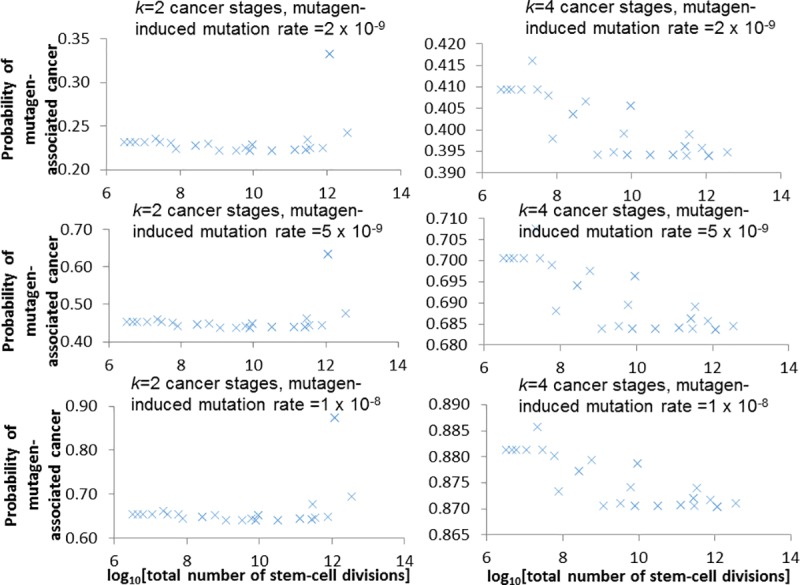
Probability [at least one mutation is mutagen-induced | cancer occurs] versus log_10_[cumulative stem cell divisions]. The conditional probability is evaluated (via expression (9)) using a generalization of the model of Wu *et al*. [11] using *k* = 2 or *k* = 4 cancer stages, a spontaneous mutation rate of 10^−8^ per cell division, and a mutagen-induced mutation rate of 2 x 10^−9^, 5 x 10^−9^ or 1 x 10^−8^ per cell division, and mutagen-associated rates increase from 0 after the first third of stem cell divisions. The data used are given in Table A1 in S1 Appendix.

The analysis of Table 3 demonstrates that in general, using the generalization of the model of Wu *et al*. [11], the probability of a cancer being mutagen-induced, Pr_*M*_, correlates significantly with the cumulative number of stem cell divisions (*p*<0.05). This correlation does not depend on the assumed value of the number of critical cancer mutations, *k*, between 1 and 4, and neither does it depend on the mutagen-assumed mutation rate. However, the correlation is slightly less significant when *k* = 2 mutations are assumed (Table 3). The trend is also shown in six particular (typical) cases in Fig 5. When *k* = 3 or *k* = 4 increasing the cumulative number of cell divisions leads to a reduction in the probability of cancer being mutagen induced, but for *k* = 1 or *k* = 2 increasing the cumulative number of cell divisions leads to an increase in the probability of cancer being mutagen induced (Table 3, Fig 5, Table A1 in S1 Appendix). As can be seen (Table 3, Fig 5, Table A1 in S1 Appendix) the correlations, although quite striking, in some cases, for example with *k* = 3 or *k* = 4, account for only a very modest variation in probability, by about 2%; the significant findings are also in some cases sensitive to removal of high-leverage datapoints (with Cook’s distance > 4 / [*n* − *p* − 1]) (Table 3).

The analyses of Tables A4 and A5 in S1 Appendix demonstrate that there are no significant correlations (*p*>0.3) between lifetime cancer-site specific population radiation risk (REIC) and the probability of that cancer being mutagen-induced, Pr_*M*_. This is the case whether the ICRP or BEIR VII recommended cancer-site specific weighting of EAR *vs* ERR models are used (Tables A4 and A5 in S1 Appendix respectively). These null results are also insensitive to the variations tested in the assumed critical number of mutations leading to cancer, *k*, in the assumed mutagen-associated mutation rate, or to removal of high-leverage datapoints (with Cook’s distance > 4 / [*n* − *p* − 1]).

**Fig 6:**
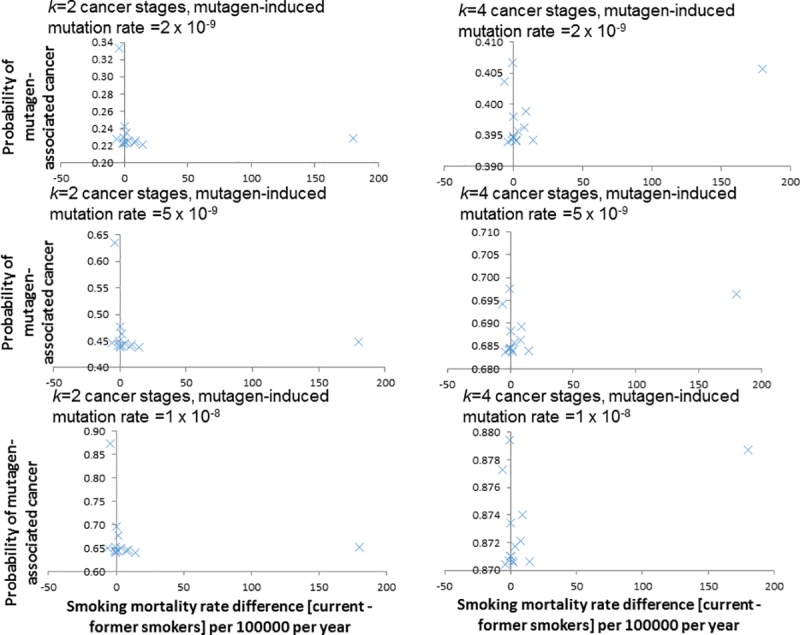
Probability [at least one mutation is mutagen-induced | cancer occurs] versus smoking-associated cancer risk (using data taken from Doll *et al*. [18]). The conditional probability is evaluated (via expression (9)) using a generalization of the model of Wu *et al*. [11] using *k* = 2 or *k* = 4 cancer stages, a spontaneous mutation rate of 10^−8^ per cell division, and a mutagen-induced mutation rate of 2 x 10^−9^, 5 x 10^−9^ or 1 x 10^−8^ per cell division, and mutagen-associated rates increase from 0 after the first third of stem cell divisions. The data used are given in Table A1 in S1 Appendix.

The analysis of Table 4 suggests that there are borderline-significant positive correlations (*p* = 0.08) between smoking-associated mortality rate difference (current vs former smokers), *Sm*_*diff*_, and Pr_*M*_. This is only the case when values of the critical number of mutations leading to cancer, *k*, is 3 or 4 and not 1 or 2), but does not strongly depend on the assumed mutagen-associated mutation rate (varying between 20–100% of the underlying rate). The trend is also shown in six particular (typical) cases in Fig 6. However, the significant findings are in all cases sensitive to removal of high-leverage points (with Cook’s distance > 4 / [*n* − *p* − 1]), resulting in loss of significance (*p*>0.3) (Table 4).

The analysis of Table 5 shows that very similar relative risks are produced to those of Table 2 if instead of the model of Wu *et al*. [11] a multistage cancer model is used. Although cancer risks span at least 23 orders of magnitude, with few exceptions relative risks generally span the range of 1–10. Little difference is made if the intermediate cells (whether stem or transit cells) that have acquired one or more mutations undergo competing processes of proliferation and differentiation/apoptosis, or if the excess mutational load falls entirely on either stem cells or transit cells (Table 5). Likewise, little difference is made if models allowing for an extra mutation for transit cells, whether in the case *k* = 2 and *k* = 3 for stem cells and transit cells respectively, or in the case *k* = 3 and *k* = 4 for stem cells and transit cells respectively (Table A6 in S1 Appendix). Fig 4 demonstrates that the relative risk tends to decrease when the value of the mutation rate multiplier, *d*, increases. This decrease is most striking when the critical number of mutations *k* = 3.

## Discussion

We have developed three separate cancer models, which share certain features, and yield similar predictions, namely substantial variation in cancer risks, by over 20 orders of magnitude (a range that is arguably highly implausible), depending on the assumed numbers of various model parameters (numbers of mutations required for cancer, mutation rates etc). However, in most cases the conditional probabilities of cancer being induced by some dominant mutagen are similar. The relative risks associated with mutagen exposure compared to background rates are also fairly stable. It was calculated that very few cancers, generally <0.5%, would arise from mutations occurring solely in stem cells rather than in a combination of stem and transit cells. However, particularly for cancers with 2 or 3 critical mutations, a substantial proportion of cancers, in some cases 100%, would have at least one mutation derived from a mutated stem cell. It should be noted that while for many common epithelial cancers of adulthood 3 or more critical mutations are plausible, the number of critical mutations may be less than this, 1 or 2, for leukemia and for childhood cancer [4]. Indeed, a recent ICRP report suggested that “it is tempting to speculate that childhood thyroid cancer requires two mutations, and a linear dose response with the short latency occurred for those carrying the pre-existing *RET/PTC* rearrangement, with radiation responsible for inducing the second hit necessary for conversion of the cells to full malignancy” [4].

We have also shown that the probability of a cancer being mutagen-induced correlates significantly (across cancer sites) with the estimated cumulative number of stem cell divisions in the associated tissue (*p*<0.05), so that at least when the number of cancer mutations, *k*, takes values of 3 or 4, values that are generally more likely than values of 1 or 2, increasing the cumulative number of cell divisions leads to a reduction in the probability of cancer being mutagen induced (Table 3, Fig 5, Table A1 in S1 Appendix). This correlation does not depend on the assumed value of the number of critical cancer mutations, or with the mutagen-associated mutation rate, in the tested range. Intuitively, one might expect a negative correlation, because when the number of stem cell divisions is very high, cancer is more likely to occur in the first third of life, before the mutagen becomes effective. Although there is indeed a negative correlation, when *k* = 3 or *k* = 4, the effect size shown in Fig 5 and Table A1 in S1 Appendix is small. Increasing the number of stem cell divisions by a factor of one million decreases the probability of mutagen-associated cancer by about 2–3%. There are also borderline significant negative correlations (*p* = 0.08) between the smoking-associated mortality rate difference (current vs former smokers) and the probability of cancer being mutagen-induced. This is only the case where values of the critical number of mutations leading to cancer, *k*, is 3 or 4, and not smaller values, of 1 or 2, but does not strongly depend on the assumed mutagen-associated mutation rate. Both of these findings provide a measure of support to the controversial findings of Tomasetti and Vogelstein [7], which suggested that cancer risk for a tissue may be associated with the total number of stem cell divisions.

We have considered a modest range in the excess dominant mutagen-associated rate, doubling the “spontaneous” mutation rate. It is probably the case that if one could subtract out all mutagens that may cause cancer a wider range of multipliers of “spontaneous” mutation rate could be considered, but it is difficult to know where one would draw the line. For example, there are endogenous mutagenic processes such as the myriad of oxidative processes within a cell that cause single (and some double) strand break damage; subtracting these off from the baseline “spontaneous” mutation rate is arguably artificial. Considering a wider range of relative increase of the “spontaneous” mutation rate would scarcely change the conclusions of the present calculations. Although in the simulations using the model of Frank *et al*. [6] (Table 1) we assume that the relative increases in mutations in the transit and stem cell populations are the same, sensitivity analysis using a special case of the multistage carcinogenesis model developed by Little *et al*. [13] in which the additional mutations were assumed to only apply either to the stem cell population or to the transit cell population (Table 5) did not suggest markedly different relative risks.

A slight limitation of the model of Frank *et al*. [6] is that stem cells and transit cells are assumed to have similar cycle times. However, by varying the mutation rates of stem and transit cells one can largely circumvent this restriction–the effect of stem cells for example having longer cycle times is essentially accounted for by the fact that mutation rates (per single division cycle) are lower for this type of cell. This model could be easily generalized to the case in which the numbers of mutations required by stem and transit cells are different, as for example might be the case in the colon if transit cells required an extra mutation to make them “sticky” to avoid being sloughed off into the lumen, as discussed by Frank *et al*. [6], although no associated calculations were carried out by them. The analysis we have conducted (Table A6 in S1 Appendix) suggests that little difference would be made by assuming that transit cells require an extra mutation from the total required to confer malignancy on stem cells.

Both the models of Frank *et al*. [6] and of Wu *et al*. [11], generalizations of which are used here, assume that stem cells never die and never fully differentiate (i.e., they never divide symmetrically into two transit cells). However, there is ample experimental evidence that stem cells can and do produce differentiated daughter cells, particularly in the intestinal crypt [39, 40], and mathematical modeling indicates that this has important effects on cancer risk [41, 42]. Many recently developed mathematical cancer models allow for such birth/death processes in the partially transformed cell compartment, although they do not generally specifically allow for this in stem and transit cells [13, 19–23]. However, the analysis we have performed (Table 5) allowing for such competing processes of cell proliferation and differentiation/apoptosis in the partially transformed stem and transit cell populations, using a special case of a multi-stage cancer model developed elsewhere [13], does not materially alter our conclusions.

All of the three models employed here assume a stem cell population that is either constant, in particular comprising a single stem cell, as in the generalization of the model of Frank *et al*. [6], or eventually constant, as in the generalization of the model of Wu *et al*. [11], or the special case of the multistage cancer model of Little *et al*. [13]. However, the precise age at which the stem cell population becomes constant differs between the three models, and would generally be largest in certain cases of the generalization of the model of Wu *et al*. [11] in which the number of stem cell divisions *n*_1_, is large in comparison with the number of transit cell divisions, *n*_2_, specifically for lung adenocarcinoma, osteosarcoma, and thyroid carcinoma. Tomasetti and Vogelstein [7] in their analysis of stem cell divisions and cancer make a similar assumption to Wu *et al*. [11], namely that after a period of symmetric stem cell division, there is a period of largely asymmetric division of stem cells into stem and transit cells; this assumption was made to derive the total number of stem cell divisions in each organ/tissue. The assumed absence of symmetric stem cell divisions in the model of Frank *et al*. [6] is arguably implausible. The model is mainly concerned with the consequences of establishment of a tissue niche from a single stem cell. As discussed by Frank *et al*. [6] it is possible that mutations associated with symmetric stem cell divisions during tissue growth and development may contribute substantially to lifetime cancer risk. As such the model of Frank *et al*. [6], although valid for study of cancers that arise in a renewing tissue such as the colon or skin in adulthood, may not be a good model of the carcinogenic process over the full lifespan.

As we make clear in the Methods the generalization of the model of Wu *et al*. [11] that we have developed allows for the mutation rates to vary depending on whether the divisions are symmetric or asymmetric. There have been a number of theoretical investigations that assess patterns of accumulation of mutational or epigenetic damage associated with asymmetric or symmetric cell divisions over the lifetime of an individual, although they do not allow for variation of mutation rate by the symmetry vs asymmetry of the division process [41, 43]. We are not aware of any data that suggest that the mutation rate per cell division varies depending on whether cell division is symmetric or asymmetric. Nevertheless this could be investigated further using this generalized model.

The generalizations of the models of Frank *et al*. [6] and Wu *et al*. [11] that we have developed assume that after a period of latency or lack of exposure, in the first third of cell divisions, when there is no effect, the mutagen acts equally at each stage of carcinogenesis. There are known to be variations between the mutagenic effect of particular agents on different stages of the carcinogenic process, so that for example ionizing radiation is thought to act at a relatively early stage, and cigarette smoke at a much later, although not final, stage [44, 45]. More generally, it is possible that mutation rates associated with endogenous (spontaneous) and exogenous (specific mutagen-associated) processes could exhibit quite complicated patterns of heterogeneity, rather than a simple early/late stage dependence, as we now discuss. There is evidence of genomic destabilization in many types of solid cancer [29], and for colon cancer the evidence is particularly strong for the involvement of chromosomal and microsatellite instability in most cancers [27, 28]. There is a large body of experimental data suggesting that transmissible genomic destabilization is associated with ionizing radiation exposure, with effects on a number of biological endpoints [25, 26]. Mutagen-associated genomic destabilization implies that the probability (per cell division) of an unaffected cell acquiring a mutation may not be the same as the probability (per cell division) of the same cell acquiring a subsequent mutation from that mutagen. As such radiation-associated genomic destabilization implies marked non-linearity in dose response, for which there is no strong evidence in the radioepidemiologic cancer literature [17, 46, 47]. This implies a minimal role for radiation-associated genomic destabilization associated with cancer induction in humans. Nevertheless, the evidence we present in Fig 4 is that with increasing levels of genomic destabilization (corresponding to larger values of the multiplier, *d*, in particular with *d* > 1) the relative risk reduces. Genomic destabilization and other similar types of non-linearity of mutagenic effect could also be modelled slightly differently than here, using generalizations of the multi-stage cancer model given here, and some others [13, 19, 48]; however these types of model would not allow for genomic stabilization, corresponding to the values of *d* < 1 in our model. The modifications required to incorporate genomic destabilization in the generalizations of the models of Frank *et al*. [6] and Wu *et al*. [11] developed here would be non-trivial.

In the colon there is abundant evidence that stem cells divide many times and remain at the base of the mucosal crypts [14, 49]. Thus, each stem cell division gives rise to one stem cell that remains at the basal location, and one transit cell. The transit cell divides a limited number of times (likely 5–9), producing more differentiating cells that move away from the basal position, maturing and eventually being terminally differentiated and sloughing off from the lumenal surface [49]. The idea that transit cells as well as stem cells could be target cells for colon cancer has been discussed previously, largely on the basis that tumors arise which are composed predominantly, or exclusively, of mucin-secreting cells, endocrine cells or even Paneth cells [50, 51]. Recently, this aspect has been emphasised, with the suggestion that in the colon adenomas likely arise from stem cells and microadenomas arise from transit amplifying cells [52]. Also, histological sections of small adenomatous polyps showed some crypts with the bottom half apparently normal and an abrupt transition to the upper mutated dysplastic half [53]. In addition, recently a small population of radioresistant Krt19 (intermediate filament keratin-19) labelled stem cells has been detected in the presumed transit cell zone of mouse colonic crypts, capable of giving rise to Lgr5^+^ stem cells with the suggestion that the former could be the target cells for colon cancer [54]. The increasing evidence for plasticity of stem and other cells in the lineage, potentially may increase the target number, especially at radiation doses which induce some cytotoxicity which transiently disturbs the homeostatic locations of particular cell types [55]. In contrast, higher doses causing more cytotoxicity may reduce the surviving target cell number. These features would add further complexity to the current models.

There is also evidence in other tissues that transit cells rather than stem cells are the more likely target. For basal cell carcinoma the normal renewal process is slower than in the colon, but the process is similar, with basal stem cells that divide and give rise to more-rapidly dividing transit lineages, each transit lineage comprising three to five rounds of cell replication [56]. A model for human skin cancer proposed that stem cells were the likely target cells for basal cell carcinomas, early progenitor cells for squamous cell cancers, and late progenitor cells for papillomas [57]. Also, there is evidence in mice that the initial radiation-induced acute myeloid leukemia (AML) stem cell may originate not only from irradiated hematopoietic stem cells (HSC), but also from multipotent and common myeloid progenitor cells [58]. In addition, in a *Cre*-knock-in mouse cancer model, lung type II cells, partially differentiated Clara cells of the terminal bronchioles, and bronchioalveolar stem cells all were identified as the cells of origin for *K-ras*-induced lung hyperplasia [59]. Interestingly, only type II cells progressed to adenocarcinoma [59].

There is a considerable body of evidence supporting the existence of what have been termed cancer stem cells (CSC), that is to say a subpopulation of cancer cells that have stem-like tumor-initiating properties. The evidence is particularly strong for leukemia [60, 61], and for colon cancer [62–65], but somewhat less for cancers of the breast [66] and brain [67]. This idea is still controversial, and the data in support of its application to certain solid cancer sites somewhat contradictory [68]. Irrespective of that, it is not clear for all cancer sites what may be the origin of the CSC. It is possible that the CSC derives from a mutated stem cell, and evidence for this is strongest for AML, where the associated CSCs have been shown to comprise distinct, hierarchically-arranged classes, similar to those observed with HSC, that dictate distinct fates [61]. It is also plausible in the light of our analysis (Table 1) since, as discussed above, AML is likely to have a small number of critical mutations. However, CSC may also arise from what we term transit cells, that already have undergone one or more stages of differentiation, via some process of de-differentiation and relocation into the stem cell niche. Although it is not known whether this does occur, in most tissues there are very many more differentiated than stem cells, and the number of steps involved in de-differentiating human adult somatic cells into pluripotent human stem cells is modest [69, 70], suggesting that this process may be more likely than the alternative, of mutation of stem cells.

Evidence has been found supporting Cairns’ hypothesis of DNA strand-segregation, which would reduce the mutation rate in the stem cell population from chronic exposures, in a number of experimental systems, including small intestinal crypts, mammary epithelium, some muscle satellite cells and progenitor cells, some central nervous system cells, although not in haematopoietic stem cells [4]. In particular, Potten *et al*. [71] using a pulse/chase experiment with tritiated thymidine (^3^HTdR), found long-term label-retaining cells in the intestinal crypts of neonatal mice. Potten *et al*. [71] hypothesized that long-term incorporation of ^3^HTdR occurred because neonatal mice have undeveloped small intestines, and that pulsing ^3^HTdR soon after the birth of the mice allowed the “immortal” DNA of adult stem cells to be labeled during their formation. These long-term (stem) cells were demonstrated to be actively cycling, as demonstrated by incorporation and release of BrdU [71]. Another mechanism for reducing the mutation rate from chronic exposures is stem cell competition for residence in the niche, between advantaged/undamaged stem cells and disadvantaged/damaged stem cells from radiation [72–74]. Presumably, in this scenario stem cells would also be advantaged over transit cells, in steady state conditions.

In summary, the analysis we have presented suggests that the probability of a cancer being mutagen-induced correlates significantly with the cumulative number of stem cell divisions, confirming an earlier report [7]; in some cases the effect is of quite modest size, and in some cases the findings are also sensitive to removal of high-leverage datapoints, but these issues do not affect the validity of the findings. Our analysis also suggests that the relative contribution to total cancer risk from mutated transit cells (as opposed solely to mutated stem cells) is relatively large, so that almost no cancers arise solely from stem-cell mutations. However, particularly for cancers with 2 or 3 critical mutations, such as leukemia, a substantial proportion of cancers, in some cases 100%, will have at least one mutation deriving from a mutated stem cell.

## Supporting information

S1 Appendix (DOCX)

S1 Supporting Information WinZip archive containing data, R scripts and output files, Excel spreadsheets, and Fortran code (*.for) and associated input (*.inp) and output (*.lis) files. (ZIP)

## References

[1] Harris H. A long view of fashions in cancer research. Bioessays. 2005;27(8):833–8. 10.1002/bies.20263 16015588

[2] United Nations Scientific Committee on the Effects of Atomic Radiation (UNSCEAR). Sources and effects of ionizing radiation. UNSCEAR 1993 report to the General Assembly, with scientific annexes. New York: United Nations; 1993. p. 1–922.

[3] Cairns J. Mutation selection and the natural history of cancer. Nature. 1975;255(5505):197–200. 1143315 10.1038/255197a0

[4] International Commission on Radiological Protection. Stem cell biology with respect to carcinogenesis aspects of radiological protection. ICRP Publication 131. Ann ICRP. 2015;44(3–4):1–357. 10.1177/0146645315595585 26637346

[5] Cairns J. Somatic stem cells and the kinetics of mutagenesis and carcinogenesis. Proc Natl Acad Sci U S A. 2002;99(16):10567–70. PubMed Central PMCID: PMCPMC124976. 10.1073/pnas.162369899 12149477 PMC124976

[6] Frank SA, Iwasa Y, Nowak MA. Patterns of cell division and the risk of cancer. Genetics. 2003;163(4):1527–32. 12702695 10.1093/genetics/163.4.1527 PMC1462514

[7] Tomasetti C, Vogelstein B. Cancer etiology. Variation in cancer risk among tissues can be explained by the number of stem cell divisions. Science. 2015;347(6217):78–81. 10.1126/science.1260825 25554788 PMC4446723

[8] O'Callaghan M. Cancer risk: accuracy of literature. Science. 2015;347(6223):729. 10.1126/science.aaa6212 25678652

[9] Little MP, Hendry JH, Puskin JS. Lack of correlation between stem-cell proliferation and radiation- or smoking-associated cancer risk. PloS one. 2016;11(3):e0150335 10.1371/journal.pone.0150335 27031507 PMC4816383

[10] Noble R, Kaltz O, Hochberg ME. Peto's paradox and human cancers. Philos Trans R Soc Lond B Biol Sci. 2015;370(1673) 20150104. PubMed Central PMCID: PMCPMC4581036. 10.1098/rstb.2015.0104 PMC4581036 26056357

[11] Wu S, Powers S, Zhu W, Hannun YA. Substantial contribution of extrinsic risk factors to cancer development. Nature. 2016;529(7584):43–7. 10.1038/nature16166 26675728 PMC4836858

[12] Watson HW, Galton F. On the probability of extinction of families. J Royal Anthropol Inst. 1875;4:138–44. Epub 1875.

[13] Little MP, Kleinerman RA, Stiller CA, Li G, Kroll ME, Murphy MFG. Analysis of retinoblastoma age incidence data using a fully stochastic cancer model. Int J Cancer. 2012;130(3):631–40. PubMed Central PMCID: PMC3167952. 10.1002/ijc.26039 21387305 PMC3167952

[14] Potten CS, Hendry JH. Radiation and Gut: Elsevier; 1995 1/1995. 312 p.

[15] Kellett M, Potten CS, Rew DA. A comparison of in vivo cell proliferation measurements in the intestine of mouse and man. Epithelial Cell Biol. 1992;1(4):147–55. 1307946

[16] Vogelstein B, Fearon ER, Hamilton SR, Kern SE, Preisinger AC, Leppert M, et al Genetic alterations during colorectal-tumor development. N Engl J Med. 1988;319(9):525–32. 10.1056/NEJM198809013190901 2841597

[17] United Nations Scientific Committee on the Effects of Atomic Radiation (UNSCEAR). UNSCEAR 2006 Report. Annex A. Epidemiological Studies of Radiation and Cancer. New York: United Nations; 2008. p. 13–322.

[18] Doll R, Peto R, Boreham J, Sutherland I. Mortality from cancer in relation to smoking: 50 years observations on British doctors. Br J Cancer. 2005;92(3):426–9. PubMed Central PMCID: PMC2362086. 10.1038/sj.bjc.6602359 15668706 PMC2362086

[19] Little MP, Vineis P, Li G. A stochastic carcinogenesis model incorporating multiple types of genomic instability fitted to colon cancer data. J Theor Biol. 2008;254(2):229–38. 10.1016/j.jtbi.2008.05.027 18640693

[20] Little MP, Wright EG. A stochastic carcinogenesis model incorporating genomic instability fitted to colon cancer data. Math Biosci. 2003;183(2):111–34. 12711407 10.1016/s0025-5564(03)00040-3

[21] Moolgavkar SH, Venzon DJ. Two-event models for carcinogenesis: incidence curves for childhood and adult tumors. Math Biosci. 1979;47(1–2):55–77.

[22] Little MP. Are two mutations sufficient to cause cancer? Some generalizations of the two-mutation model of carcinogenesis of Moolgavkar, Venzon, and Knudson, and of the multistage model of Armitage and Doll. Biometrics. 1995;51(4):1278–91. 8589222

[23] Little MP, Li G. Stochastic modelling of colon cancer: is there a role for genomic instability? Carcinogenesis. 2007;28(2):479–87. 10.1093/carcin/bgl173 16973671

[24] Little MP, Haylock RGE, Muirhead CR. Modelling lung tumour risk in radon-exposed uranium miners using generalizations of the two-mutation model of Moolgavkar, Venzon and Knudson. Int J Radiat Biol. 2002;78(1):49–68. 10.1080/09553000110085797 11747553

[25] Morgan WF. Non-targeted and delayed effects of exposure to ionizing radiation: I. Radiation-induced genomic instability and bystander effects in vitro. Radiat Res. 2003;159(5):567–80. 12710868 10.1667/0033-7587(2003)159[0567:nadeoe]2.0.co;2

[26] Morgan WF. Non-targeted and delayed effects of exposure to ionizing radiation: II. Radiation-induced genomic instability and bystander effects in vivo, clastogenic factors and transgenerational effects. Radiat Res. 2003;159(5):581–96. 12710869 10.1667/0033-7587(2003)159[0581:nadeoe]2.0.co;2

[27] Cisyk AL, Penner-Goeke S, Lichtensztejn Z, Nugent Z, Wightman RH, Singh H, et al Characterizing the prevalence of chromosome instability in interval colorectal cancer. Neoplasia. 2015;17(3):306–16. PubMed Central PMCID: PMCPMC4372653. 10.1016/j.neo.2015.02.001 25810015 PMC4372653

[28] Worthley DL, Leggett BA. Colorectal cancer: molecular features and clinical opportunities. Clin Biochem Rev. 2010;31(2):31–8. PubMed Central PMCID: PMCPMC2874430. 20498827 PMC2874430

[29] Lengauer C, Kinzler KW, Vogelstein B. Genetic instabilities in human cancers. Nature. 1998;396(6712):643–9. 10.1038/25292 9872311

[30] Preston DL, Ron E, Tokuoka S, Funamoto S, Nishi N, Soda M, et al Solid cancer incidence in atomic bomb survivors: 1958–1998. RadiatRes. 2007;168(1):1–64. 10.1667/RR0763.1 17722996

[31] International Commission on Radiological Protection. The 2007 Recommendations of the International Commission on Radiological Protection. ICRP publication 103. Ann ICRP. 2007;37(2–4):1–332. 10.1016/j.icrp.2007.10.003 18082557

[32] Committee to Assess Health Risks from Exposure to Low Levels of Ionizing Radiation NRC Health Risks from Exposure to Low Levels of Ionizing Radiation: BEIR VII—Phase 2. Washington, DC, USA: National Academy Press; 2006. 1–406 p. 25077203

[33] Jacob P, Rühm W, Walsh L, Blettner M, Hammer G, Zeeb H. Is cancer risk of radiation workers larger than expected? Occup Environ Med. 2009;66(12):789–96. 10.1136/oem.2008.043265 19570756 PMC2776242

[34] Rühm W, Azizova TV, Bouffler SD, Little MP, Shore RE, Walsh L, et al Dose-rate effects in radiation biology and radiation protection. Ann ICRP. 2016;45(1 supp.):262–79. 26960819 10.1177/0146645316629336

[35] Rao CR. Linear statistical inference and its applications. 2nd edition Singapore: John Wiley & Sons, Inc; 2002. 1–625 p.

[36] R Project version 3.2.2. R version 3.2.2 http://www.r-project.org/. Comprehensive R Archive Network (CRAN); 2015.

[37] Cook RD. Detection of influential observation in linear regression. Technometrics. 1977;19(1):15–8.

[38] Bollen KA, Jackman RW. Regression diagnostics: An expository treatment of outliers and influential cases In: Fox J, Long JS, editors. Modern Methods of Data Analysis Newbury Park, CA, USA: Sage; 1990 p. 257–91.

[39] Morrison SJ, Kimble J. Asymmetric and symmetric stem-cell divisions in development and cancer. Nature. 2006;441(7097):1068–74. 10.1038/nature04956 16810241

[40] Snippert HJ, van der Flier LG, Sato T, van Es JH, van den Born M, Kroon-Veenboer C, et al Intestinal crypt homeostasis results from neutral competition between symmetrically dividing Lgr5 stem cells. Cell. 2010;143(1):134–44. 10.1016/j.cell.2010.09.016 20887898

[41] Shahriyari L, Komarova NL. Symmetric vs. asymmetric stem cell divisions: an adaptation against cancer? PloS one. 2013;8(10):e76195 PubMed Central PMCID: PMCPMC3812169. 10.1371/journal.pone.0076195 24204602 PMC3812169

[42] Yang J, Plikus MV, Komarova NL. The Role of Symmetric Stem Cell Divisions in Tissue Homeostasis. PLoS Comput Biol. 2015;11(12):e1004629 PubMed Central PMCID: PMCPMC4689538. 10.1371/journal.pcbi.1004629 26700130 PMC4689538

[43] McHale PT, Lander AD. The protective role of symmetric stem cell division on the accumulation of heritable damage. PLoS Comput Biol. 2014;10(8):e1003802 PubMed Central PMCID: PMCPMC4133021. 10.1371/journal.pcbi.1003802 25121484 PMC4133021

[44] Peto R. Epidemiology, multistage models, and short-term mutagenicity tests In: Hiatt HH, Winsten JA, editors. Origins of human cancer. Cold Spring Harbor: Cold Spring Harbor Laboratory; 1977 p. 1403–28.

[45] Breslow NE, Day NE. Statistical methods in cancer research. Volume II—The design and analysis of cohort studies. IARC SciPubl. 1987;(82):1–406. 3329634

[46] Little MP, Wakeford R, Tawn EJ, Bouffler SD, Berrington de Gonzalez A. Risks associated with low doses and low dose rates of ionizing radiation: why linearity may be (almost) the best we can do. Radiology. 2009;251(1):6–12. 10.1148/radiol.2511081686 19332841 PMC2663578

[47] Doss M, Little MP, Orton CG. Point/Counterpoint: low-dose radiation is beneficial, not harmful. Med Phys. 2014;41(7):070601 10.1118/1.4881095 24989368 PMC4109571

[48] Little MP. Cancer models, genomic instability and somatic cellular Darwinian evolution. Biol Direct. 2010;5:19 10.1186/1745-6150-5-19 20406436 PMC2873266

[49] Bach SP, Renehan AG, Potten CS. Stem cells: the intestinal stem cell as a paradigm. Carcinogenesis. 2000;21(3):469–76. 10688867 10.1093/carcin/21.3.469

[50] Pierce GB, Speers WC. Tumors as caricatures of the process of tissue renewal: prospects for therapy by directing differentiation. Cancer Res. 1988;48(8):1996–2004. 2450643

[51] Humphries A, Wright NA. Colonic crypt organization and tumorigenesis. Nat Rev Cancer. 2008;8(6):415–24. 10.1038/nrc2392 18480839

[52] Huels DJ, Sansom OJ. Stem vs non-stem cell origin of colorectal cancer. Br J Cancer. 2015;113(1):1–5. PubMed Central PMCID: PMCPMC4647531. 10.1038/bjc.2015.214 26110974 PMC4647531

[53] Shih IM, Wang TL, Traverso G, Romans K, Hamilton SR, Ben-Sasson S, et al Top-down morphogenesis of colorectal tumors. Proc Natl Acad Sci U S A. 2001;98(5):2640–5. 10.1073/pnas.051629398 11226292 PMC30191

[54] Asfaha S, Hayakawa Y, Muley A, Stokes S, Graham TA, Ericksen RE, et al Krt19(+)/Lgr5(-) Cells Are Radioresistant Cancer-Initiating Stem Cells in the Colon and Intestine. Cell Stem Cell. 2015;16(6):627–38. PubMed Central PMCID: PMCPMC4457942. 10.1016/j.stem.2015.04.013 26046762 PMC4457942

[55] Hendry JH, Otsuka K. The role of gene mutations and gene products in intestinal tissue reactions from ionising radiation. Mutat Res. 2016;770(Pt B):328–39. 10.1016/j.mrrev.2016.07.006 27919339

[56] Janes SM, Lowell S, Hutter C. Epidermal stem cells. J Pathol. 2002;197(4):479–91. 10.1002/path.1156 12115864

[57] Sell S. Stem cell origin of cancer and differentiation therapy. Crit Rev Oncol Hematol. 2004;51(1):1–28. 10.1016/j.critrevonc.2004.04.007 15207251

[58] Olme CH, Brown N, Finnon R, Bouffler SD, Badie C. Frequency of acute myeloid leukaemia-associated mouse chromosome 2 deletions in X-ray exposed immature haematopoietic progenitors and stem cells. Mutat Res. 2013;756(1–2):119–26. PubMed Central PMCID: PMC4028086. 10.1016/j.mrgentox.2013.04.018 23665297 PMC4028086

[59] Xu X, Rock JR, Lu Y, Futtner C, Schwab B, Guinney J, et al Evidence for type II cells as cells of origin of K-Ras-induced distal lung adenocarcinoma. Proc Natl Acad Sci U S A. 2012;109(13):4910–5. PubMed Central PMCID: PMCPMC3323959. 10.1073/pnas.1112499109 22411819 PMC3323959

[60] Bonnet D, Dick JE. Human acute myeloid leukemia is organized as a hierarchy that originates from a primitive hematopoietic cell. Nat Med. 1997;3(7):730–7. 9212098 10.1038/nm0797-730

[61] Hope KJ, Jin L, Dick JE. Acute myeloid leukemia originates from a hierarchy of leukemic stem cell classes that differ in self-renewal capacity. Nat Immunol. 2004;5(7):738–43. 10.1038/ni1080 15170211

[62] Vermeulen L, Todaro M, de Sousa Mello F, Sprick MR, Kemper K, Perez Alea M, et al Single-cell cloning of colon cancer stem cells reveals a multi-lineage differentiation capacity. Proc Natl Acad Sci U S A. 2008;105(36):13427–32. PubMed Central PMCID: PMCPMC2533206. 10.1073/pnas.0805706105 18765800 PMC2533206

[63] Odoux C, Fohrer H, Hoppo T, Guzik L, Stolz DB, Lewis DW, et al A stochastic model for cancer stem cell origin in metastatic colon cancer. Cancer Res. 2008;68(17):6932–41. PubMed Central PMCID: PMCPMC2562348. 10.1158/0008-5472.CAN-07-5779 18757407 PMC2562348

[64] Todaro M, Alea MP, Di Stefano AB, Cammareri P, Vermeulen L, Iovino F, et al Colon cancer stem cells dictate tumor growth and resist cell death by production of interleukin-4. Cell Stem Cell. 2007;1(4):389–402. 10.1016/j.stem.2007.08.001 18371377

[65] Chu P, Clanton DJ, Snipas TS, Lee J, Mitchell E, Nguyen ML, et al Characterization of a subpopulation of colon cancer cells with stem cell-like properties. Int J Cancer. 2009;124(6):1312–21. 10.1002/ijc.24061 19072981

[66] Al-Hajj M, Wicha MS, Benito-Hernandez A, Morrison SJ, Clarke MF. Prospective identification of tumorigenic breast cancer cells. Proc Natl Acad Sci U S A. 2003;100(7):3983–8. PubMed Central PMCID: PMCPMC153034. 10.1073/pnas.0530291100 12629218 PMC153034

[67] Singh SK, Clarke ID, Terasaki M, Bonn VE, Hawkins C, Squire J, et al Identification of a cancer stem cell in human brain tumors. Cancer Res. 2003;63(18):5821–8. 14522905

[68] Hill RP. Identifying cancer stem cells in solid tumors: case not proven. Cancer Res. 2006;66(4):1891–5; discussion 0. 10.1158/0008-5472.CAN-05-3450 16488984

[69] Takahashi K, Tanabe K, Ohnuki M, Narita M, Ichisaka T, Tomoda K, et al Induction of pluripotent stem cells from adult human fibroblasts by defined factors. Cell. 2007;131(5):861–72. 10.1016/j.cell.2007.11.019 18035408

[70] Yu J, Vodyanik MA, Smuga-Otto K, Antosiewicz-Bourget J, Frane JL, Tian S, et al Induced pluripotent stem cell lines derived from human somatic cells. Science. 2007;318(5858):1917–20. 10.1126/science.1151526 18029452

[71] Potten CS, Owen G, Booth D. Intestinal stem cells protect their genome by selective segregation of template DNA strands. J Cell Sci. 2002;115(Pt 11):2381–8. 12006622 10.1242/jcs.115.11.2381

[72] Niwa O. Roles of stem cells in tissue turnover and radiation carcinogenesis. Radiat Res. 2010;174(6):833–9. 10.1667/RR1970.1 21128807

[73] Vermeulen L, Morrissey E, van der Heijden M, Nicholson AM, Sottoriva A, Buczacki S, et al Defining stem cell dynamics in models of intestinal tumor initiation. Science. 2013;342(6161):995–8. 10.1126/science.1243148 24264992

[74] Snippert HJ, Schepers AG, van Es JH, Simons BD, Clevers H. Biased competition between Lgr5 intestinal stem cells driven by oncogenic mutation induces clonal expansion. EMBO Rep. 2014;15(1):62–9. PubMed Central PMCID: PMCPMC3983678. 10.1002/embr.201337799 24355609 PMC3983678

