# Supplementary material for: Mathematical models of tissue stem and transit target cell divisions and the risk of radiation- or smoking-associated cancer
Source: PLoS Comput Biol. 2017 Feb 14;13(2):e1005391. doi: 10.1371/journal.pcbi.1005391 (PMC5347390; doi:10.1371/journal.pcbi.1005391)
Supplement: S1 Appendix — (DOCX) [file pcbi.1005391.s001.docx]

# S1 Appendix

# Table A1 in S1 Appendix. log_10_[divisions of all stem cells over a lifetime] and conditional probability[at least one mutation is mutagen-induced [cancer occurs]. The conditional probability is evaluated (via expression (9)) using a generalization of the model of Wu *et al.* [1], assuming various given numbers of symmetric stem-cell divisions ($n_{1}$ ), asymmetric divisions ($n_{2}$) (per endpoint), and mutagen-induced mutation rates ($u_{M}$ ), and mutagen-associated rates increase from 0 after the first third of stem cell divisions. A spontaneous mutation rate per cell division of $u_{S}=10^{-8}$ is used throughout.

|  | log_10_[divisions of all stem cells over lifetime] | Number of symmetric (stem-cell) divisions (*n*_1_) | Number of asymmetric divisions (*n*_2_) | Conditional probability[at least one mutation is mutagen-induced | cancer occurs] | | | | | | | | | | | |
| --- | --- | --- | --- | --- | --- | --- | --- | --- | --- | --- | --- | --- | --- | --- | --- |
| Number of cancer stages (*k*) |  |  |  | *k* = 1 | *k* = 2 | *k* = 3 | *k* = 4 | *k* = 1 | *k* = 2 | *k* = 3 | *k* = 4 | *k* = 1 | *k* = 2 | *k* = 3 | *k* = 4 |
| Mutagen-induced mutation rate per cell division (*u_M_*) | |  |  | 2 x 10^-9^ | 2 x 10^-9^ | 2 x 10^-9^ | 2 x 10^-9^ | 5 x 10^-9^ | 5 x 10^-9^ | 5 x 10^-9^ | 5 x 10^-9^ | 1 x 10^-8^ | 1 x 10^-8^ | 1 x 10^-8^ | 1 x 10^-8^ |
| Acute myeloid leukemia | 11.11 | 27 | 960 | 1.0000 | 0.2232 | 0.3134 | 0.3943 | 1.0000 | 0.4408 | 0.5786 | 0.6841 | 1.0000 | 0.6446 | 0.7844 | 0.8707 |
| Basal cell carcinoma | 12.55 | 32 | 608 | 1.0000 | 0.2419 | 0.3138 | 0.3948 | 1.0000 | 0.4771 | 0.5791 | 0.6846 | 1.0000 | 0.6957 | 0.7848 | 0.8710 |
| Chronic lymphocytic leukemia | 11.11 | 27 | 960 | 1.0000 | 0.2232 | 0.3134 | 0.3943 | 1.0000 | 0.4408 | 0.5786 | 0.6841 | 1.0000 | 0.6446 | 0.7844 | 0.8707 |
| Colorectal adenocarcinoma | 12.07 | 28 | 5840 | 1.0000 | 0.3327 | 0.3131 | 0.3939 | 1.0000 | 0.6357 | 0.5782 | 0.6837 | 1.0000 | 0.8737 | 0.7841 | 0.8704 |
| Colorectal adenocarcinoma with FAP | 12.07 | 28 | 5840 | 1.0000 | 0.3327 | 0.3131 | 0.3939 | 1.0000 | 0.6357 | 0.5782 | 0.6837 | 1.0000 | 0.8737 | 0.7841 | 0.8704 |
| Colorectal adenocarcinoma with Lynch syndrome | 12.07 | 28 | 5840 | 1.0000 | 0.3327 | 0.3131 | 0.3939 | 1.0000 | 0.6357 | 0.5782 | 0.6837 | 1.0000 | 0.8737 | 0.7841 | 0.8704 |
| Duodenum adenocarcinoma | 9.89 | 22 | 1947 | 1.0000 | 0.2218 | 0.3133 | 0.3942 | 1.0000 | 0.4381 | 0.5784 | 0.6839 | 1.0000 | 0.6408 | 0.7843 | 0.8706 |
| Duodenum adenocarcinoma with FAP | 9.89 | 22 | 1947 | 1.0000 | 0.2218 | 0.3133 | 0.3942 | 1.0000 | 0.4381 | 0.5784 | 0.6839 | 1.0000 | 0.6408 | 0.7843 | 0.8706 |
| Esophageal squamous cell carcinoma | 9.08 | 20 | 1390 | 0.8610 | 0.2217 | 0.3133 | 0.3942 | 0.9928 | 0.4378 | 0.5785 | 0.6839 | 0.9999 | 0.6404 | 0.7843 | 0.8706 |
| Gallbladder non-papillary adenocarcinoma | 7.89 | 21 | 47 | 0.2188 | 0.2241 | 0.3166 | 0.3980 | 0.4493 | 0.4416 | 0.5827 | 0.6882 | 0.6813 | 0.6442 | 0.7878 | 0.8734 |
| Glioblastoma | 8.43 | 27 | 2 | 0.9953 | 0.2277 | 0.3213 | 0.4036 | 1.0000 | 0.4471 | 0.5889 | 0.6943 | 1.0000 | 0.6497 | 0.7927 | 0.8773 |
| Head & neck squamous cell carcinoma | 10.50 | 24 | 1720 | 1.0000 | 0.2222 | 0.3133 | 0.3942 | 1.0000 | 0.4389 | 0.5785 | 0.6840 | 1.0000 | 0.6420 | 0.7843 | 0.8706 |
| Head & neck squamous cell carcinoma with HPV-16 | 10.50 | 24 | 1720 | 1.0000 | 0.2222 | 0.3133 | 0.3942 | 1.0000 | 0.4389 | 0.5785 | 0.6840 | 1.0000 | 0.6420 | 0.7843 | 0.8706 |
| Hepatocellular carcinoma | 11.43 | 31 | 88 | 1.0000 | 0.2233 | 0.3151 | 0.3963 | 1.0000 | 0.4405 | 0.5808 | 0.6862 | 1.0000 | 0.6434 | 0.7862 | 0.8721 |
| Hepatocellular carcinoma with HCV | 11.43 | 31 | 88 | 1.0000 | 0.2233 | 0.3151 | 0.3963 | 1.0000 | 0.4405 | 0.5808 | 0.6862 | 1.0000 | 0.6434 | 0.7862 | 0.8721 |
| Lung adenocarcinoma (nonsmokers) | 9.97 | 30 | 6 | 1.0000 | 0.2290 | 0.3231 | 0.4056 | 1.0000 | 0.4491 | 0.5910 | 0.6964 | 1.0000 | 0.6518 | 0.7944 | 0.8787 |
| Lung adenocarcinoma (smokers) | 9.97 | 30 | 6 | 1.0000 | 0.2290 | 0.3231 | 0.4056 | 1.0000 | 0.4491 | 0.5910 | 0.6964 | 1.0000 | 0.6518 | 0.7944 | 0.8787 |
| Medulloblastoma | 8.43 | 27 | 2 | 0.9953 | 0.2277 | 0.3213 | 0.4036 | 1.0000 | 0.4471 | 0.5889 | 0.6943 | 1.0000 | 0.6497 | 0.7927 | 0.8773 |
| Melanoma | 11.88 | 32 | 199 | 1.0000 | 0.2252 | 0.3146 | 0.3957 | 1.0000 | 0.4444 | 0.5802 | 0.6856 | 1.0000 | 0.6492 | 0.7857 | 0.8717 |
| Osteosarcoma | 7.47 | 22 | 5 | 0.2031 | 0.2315 | 0.3263 | 0.4095 | 0.4194 | 0.4528 | 0.5952 | 0.7006 | 0.6426 | 0.6555 | 0.7978 | 0.8813 |
| Osteosarcoma of the arms | 6.66 | 19 | 5 | 0.2031 | 0.2315 | 0.3263 | 0.4095 | 0.4194 | 0.4528 | 0.5952 | 0.7006 | 0.6426 | 0.6555 | 0.7978 | 0.8813 |
| Osteosarcoma of the head | 6.78 | 20 | 5 | 0.2031 | 0.2315 | 0.3263 | 0.4095 | 0.4194 | 0.4528 | 0.5952 | 0.7006 | 0.6426 | 0.6555 | 0.7978 | 0.8813 |
| Osteosarcoma of the legs | 7.05 | 21 | 5 | 0.2031 | 0.2315 | 0.3263 | 0.4095 | 0.4194 | 0.4528 | 0.5952 | 0.7006 | 0.6426 | 0.6555 | 0.7978 | 0.8813 |
| Osteosarcoma of the pelvis | 6.50 | 19 | 5 | 0.2031 | 0.2315 | 0.3263 | 0.4095 | 0.4194 | 0.4528 | 0.5952 | 0.7006 | 0.6426 | 0.6555 | 0.7978 | 0.8813 |
| Ovarian germ cell | 7.34 | 23 | 2 | 0.2867 | 0.2359 | 0.3321 | 0.4162 | 0.5624 | 0.4593 | 0.6025 | 0.7077 | 0.8008 | 0.6620 | 0.8035 | 0.8857 |
| Pancreatic ductal adenocarcinoma | 11.54 | 32 | 80 | 1.0000 | 0.2253 | 0.3174 | 0.3989 | 1.0000 | 0.4437 | 0.5837 | 0.6892 | 1.0000 | 0.6468 | 0.7886 | 0.8740 |
| Pancreatic endocrine (islet cell) carcinoma | 9.78 | 26 | 80 | 0.9999 | 0.2249 | 0.3176 | 0.3992 | 1.0000 | 0.4428 | 0.5840 | 0.6895 | 1.0000 | 0.6454 | 0.7888 | 0.8742 |
| Small intestine adenocarcinoma | 11.47 | 27 | 2920 | 1.0000 | 0.2347 | 0.3132 | 0.3941 | 1.0000 | 0.4633 | 0.5783 | 0.6838 | 1.0000 | 0.6769 | 0.7842 | 0.8705 |
| Testicular germ cell cancer | 9.52 | 23 | 463 | 0.9957 | 0.2220 | 0.3138 | 0.3947 | 1.0000 | 0.4384 | 0.5791 | 0.6846 | 1.0000 | 0.6410 | 0.7848 | 0.8710 |
| Thyroid papillary/follicular carcinoma | 8.77 | 26 | 7 | 0.9544 | 0.2297 | 0.3239 | 0.4067 | 0.9996 | 0.4501 | 0.5922 | 0.6976 | 1.0000 | 0.6527 | 0.7954 | 0.8794 |
| Thyroid medullary carcinoma | 7.77 | 23 | 7 | 0.3148 | 0.2305 | 0.3250 | 0.4079 | 0.6058 | 0.4513 | 0.5936 | 0.6989 | 0.8399 | 0.6540 | 0.7964 | 0.8803 |

Table A2 in S1 Appendix. Probabilities of cancer for various sites, conditional probability of a cancer being mutagen induced (expression (9)), and the relative risk (expression (10)), assuming a spontaneous mutation rate ($u_{S}$) = 10^-6^ per cell division, and *k*=2 to 4 critical cancer genes, mutagen-associated rates increase from 0 after the first third of stem cell divisions, using a generalization of the model of Wu *et al.* [1]. Assumptions as to the number of symmetric ($n_{1}$) and asymmetric cell divisions ($n_{2}$) are as for the paper of Wu *et al.* [1].

| Cancer site | Mutagen-induced mutation rate per cell division ($u_{M}$) after first third of stem-cell divisions | *k*=2 | | |  | *k*=3 | | |  | *k*=4 | | |
| --- | --- | --- | --- | --- | --- | --- | --- | --- | --- | --- | --- | --- |
|  |  | Total probability of cancer | Relative risk | Pr[at least one mutation is mutagen-induced\|cancer occurs] (%) |  | Total probability of cancer | Relative risk | Pr[at least one mutation is mutagen-induced\|cancer occurs] (%) |  | Total probability of cancer | Relative risk | Pr[at least one mutation is mutagen-induced\|cancer occurs] (%) |
| Acute myeloid leukemia  ($n_{1}=27,n_{2}=960$) | 0 | 1.00 | 1.00 | 0.0 |  | 1.21 x 10^-1^ | 1.00 | 0.0 |  | 1.27 x 10^-4^ | 1.00 | 0.0 |
|  | 2 x 10^-7^ | 1.00 | 1.00 | 100.0 |  | 1.71 x 10^-1^ | 1.42 | 33.4 |  | 2.10 x 10^-4^ | 1.65 | 39.4 |
|  | 5 x 10^-7^ | 1.00 | 1.00 | 100.0 |  | 2.63 x 10^-1^ | 2.18 | 61.5 |  | 4.02 x 10^-4^ | 3.16 | 68.4 |
|  | 1 x 10^-6^ | 1.00 | 1.00 | 100.0 |  | 4.50 x 10^-1^ | 3.72 | 83.2 |  | 9.81 x 10^-4^ | 7.72 | 87.1 |
| Basal cell carcinoma  ($n_{1}=32,n_{2}=608$) | 0 | 1.00 | 1.00 | 0.0 |  | 6.75 x 10^-1^ | 1.00 | 0.0 |  | 7.19 x 10^-4^ | 1.00 | 0.0 |
|  | 2 x 10^-7^ | 1.00 | 1.00 | 100.0 |  | 8.06 x 10^-1^ | 1.19 | 49.9 |  | 1.19 x 10^-3^ | 1.65 | 39.5 |
|  | 5 x 10^-7^ | 1.00 | 1.00 | 100.0 |  | 9.31 x 10^-1^ | 1.38 | 84.6 |  | 2.28 x 10^-3^ | 3.17 | 68.5 |
|  | 1 x 10^-6^ | 1.00 | 1.00 | 100.0 |  | 9.95 x 10^-1^ | 1.47 | 98.9 |  | 5.56 x 10^-3^ | 7.73 | 87.1 |
| Colorectal adenocarcinoma  ($n_{1}=28,n_{2}=5840$) | 0 | 1.00 | 1.00 | 0.0 |  | 1.00 | 1.00 | 0.0 |  | 2.70 x 10^-1^ | 1.00 | 0.0 |
|  | 2 x 10^-7^ | 1.00 | 1.00 | 100.0 |  | 1.00 | 1.00 | 100.0 |  | 4.04 x 10^-1^ | 1.50 | 45.6 |
|  | 5 x 10^-7^ | 1.00 | 1.00 | 100.0 |  | 1.00 | 1.00 | 100.0 |  | 6.29 x 10^-1^ | 2.33 | 78.2 |
|  | 1 x 10^-6^ | 1.00 | 1.00 | 100.0 |  | 1.00 | 1.00 | 100.0 |  | 9.10 x 10^-1^ | 3.37 | 96.4 |
| Esophageal squamous cell carcinoma  ($n_{1}=20,n_{2}=1390$) | 0 | 8.75 x 10^-1^ | 1.00 | 0.0 |  | 2.93 x 10^-3^ | 1.00 | 0.0 |  | 4.13 x 10^-6^ | 1.00 | 0.0 |
|  | 2 x 10^-7^ | 9.31 x 10^-1^ | 1.06 | 48.0 |  | 4.26 x 10^-3^ | 1.45 | 31.4 |  | 6.82 x 10^-6^ | 1.65 | 39.4 |
|  | 5 x 10^-7^ | 9.75 x 10^-1^ | 1.11 | 82.2 |  | 6.93 x 10^-3^ | 2.37 | 57.9 |  | 1.31 x 10^-5^ | 3.16 | 68.4 |
|  | 1 x 10^-6^ | 9.97 x 10^-1^ | 1.14 | 97.8 |  | 1.35 x 10^-2^ | 4.60 | 78.5 |  | 3.19 x 10^-5^ | 7.71 | 87.1 |
| Lung adenocarcinoma  ($n_{1}=30,n_{2}=6$) | 0 | 7.51 x 10^-1^ | 1.00 | 0.0 |  | 5.01 x 10^-5^ | 1.00 | 0.0 |  | 1.80 x 10^-9^ | 1.00 | 0.0 |
|  | 2 x 10^-7^ | 8.36 x 10^-1^ | 1.11 | 40.5 |  | 7.40 x 10^-5^ | 1.48 | 32.3 |  | 3.03 x 10^-9^ | 1.68 | 40.6 |
|  | 5 x 10^-7^ | 9.20 x 10^-1^ | 1.22 | 73.7 |  | 1.22 x 10^-4^ | 2.45 | 59.1 |  | 5.94 x 10^-9^ | 3.29 | 69.6 |
|  | 1 x 10^-6^ | 9.82 x 10^-1^ | 1.31 | 94.3 |  | 2.44 x 10^-4^ | 4.86 | 79.4 |  | 1.49 x 10^-8^ | 8.24 | 87.9 |
| Osteosarcoma  ($n_{1}=22,n_{2}=5$) | 0 | 3.05 x 10^-3^ | 1.00 | 0.0 |  | 8.26 x 10^-8^ | 1.00 | 0.0 |  | 2.23 x 10^-12^ | 1.00 | 0.0 |
|  | 2 x 10^-7^ | 3.97 x 10^-3^ | 1.30 | 23.2 |  | 1.23 x 10^-7^ | 1.48 | 32.6 |  | 3.77 x 10^-12^ | 1.69 | 40.9 |
|  | 5 x 10^-7^ | 5.57 x 10^-3^ | 1.83 | 45.3 |  | 2.04 x 10^-7^ | 2.47 | 59.5 |  | 7.44 x 10^-12^ | 3.34 | 70.1 |
|  | 1 x 10^-6^ | 8.84 x 10^-3^ | 2.89 | 65.6 |  | 4.08 x 10^-7^ | 4.95 | 79.8 |  | 1.88 x 10^-11^ | 8.42 | 88.1 |
| Thyroid papillary/follicular carcinoma  ($n_{1}=26,n_{2}=7$) | 0 | 7.05 x 10^-2^ | 1.00 | 0.0 |  | 2.41 x 10^-6^ | 1.00 | 0.0 |  | 7.96 x 10^-11^ | 1.00 | 0.0 |
|  | 2 x 10^-7^ | 9.05 x 10^-2^ | 1.28 | 23.8 |  | 3.57 x 10^-6^ | 1.48 | 32.4 |  | 1.34 x 10^-10^ | 1.69 | 40.7 |
|  | 5 x 10^-7^ | 1.24 x 10^-1^ | 1.77 | 46.7 |  | 5.91 x 10^-6^ | 2.45 | 59.2 |  | 2.63 x 10^-10^ | 3.31 | 69.8 |
|  | 1 x 10^-6^ | 1.90 x 10^-1^ | 2.69 | 67.6 |  | 1.18 x 10^-5^ | 4.89 | 79.5 |  | 6.60 x 10^-10^ | 8.29 | 87.9 |

Table A3 in S1 Appendix. Probabilities of cancer for various sites, conditional probability of a cancer being mutagen induced (expression (9)), and the relative risk (expression (10)), assuming a spontaneous mutation rate ($u_{S}$) = 10^-8^ per cell division, and *k*=2 to 4 critical cancer genes, mutagen-associated rates increase from 0 at birth, using a generalization of the model of Wu *et al.* [1]. Assumptions as to the number of symmetric ($n_{1}$) and asymmetric cell divisions ($n_{2}$) are as for the paper of Wu *et al.* [1].

| Cancer site | Mutagen-induced mutation rate per cell division ($u_{M}$) | *k*=2 | | |  | *k*=3 | | |  | *k*=4 | | |
| --- | --- | --- | --- | --- | --- | --- | --- | --- | --- | --- | --- | --- |
|  |  | Total probability of cancer | Relative risk | Pr[at least one mutation is mutagen-induced\|cancer occurs] (%) |  | Total probability of cancer | Relative risk | Pr[at least one mutation is mutagen-induced\|cancer occurs] (%) |  | Total probability of cancer | Relative risk | Pr[at least one mutation is mutagen-induced\|cancer occurs] (%) |
| Acute myeloid leukemia  ($n_{1}=27,n_{2}=960$) | 0 | 1.30 x 10^-2^ | 1.00 | 0.0 |  | 1.29 x 10^-7^ | 1.00 | 0.0 |  | 1.27 x 10^-12^ | 1.00 | 0.0 |
|  | 2 x 10^-9^ | 1.87 x 10^-2^ | 1.44 | 30.8 |  | 2.23 x 10^-7^ | 1.73 | 42.1 |  | 2.64 x 10^-12^ | 2.07 | 51.8 |
|  | 5 x 10^-9^ | 2.90 x 10^-2^ | 2.23 | 55.9 |  | 4.36 x 10^-7^ | 3.37 | 70.4 |  | 6.45 x 10^-12^ | 5.06 | 80.2 |
|  | 1 x 10^-8^ | 5.10 x 10^-2^ | 3.92 | 75.5 |  | 1.03 x 10^-6^ | 8.00 | 87.5 |  | 2.04 x 10^-11^ | 16.00 | 93.8 |
| Basal cell carcinoma  ($n_{1}=32,n_{2}=608$) | 0 | 1.61 x 10^-1^ | 1.00 | 0.0 |  | 1.13 x 10^-6^ | 1.00 | 0.0 |  | 7.21 x 10^-12^ | 1.00 | 0.0 |
|  | 2 x 10^-9^ | 2.24 x 10^-1^ | 1.39 | 33.3 |  | 1.95 x 10^-6^ | 1.73 | 42.1 |  | 1.49 x 10^-11^ | 2.07 | 51.8 |
|  | 5 x 10^-9^ | 3.27 x 10^-1^ | 2.03 | 60.4 |  | 3.80 x 10^-6^ | 3.37 | 70.4 |  | 3.65 x 10^-11^ | 5.06 | 80.2 |
|  | 1 x 10^-8^ | 5.05 x 10^-1^ | 3.13 | 81.2 |  | 9.01 x 10^-6^ | 8.00 | 87.5 |  | 1.15 x 10^-10^ | 16.00 | 93.8 |
| Colorectal adenocarcinoma  ($n_{1}=28,n_{2}=5840$) | 0 | 6.03 x 10^-1^ | 1.00 | 0.0 |  | 5.42 x 10^-5^ | 1.00 | 0.0 |  | 3.18 x 10^-9^ | 1.00 | 0.0 |
|  | 2 x 10^-9^ | 7.36 x 10^-1^ | 1.22 | 45.4 |  | 9.37 x 10^-5^ | 1.73 | 42.1 |  | 6.60 x 10^-9^ | 2.07 | 51.8 |
|  | 5 x 10^-9^ | 8.75 x 10^-1^ | 1.45 | 78.3 |  | 1.83 x 10^-4^ | 3.37 | 70.4 |  | 1.61 x 10^-8^ | 5.06 | 80.2 |
|  | 1 x 10^-8^ | 9.75 x 10^-1^ | 1.62 | 96.1 |  | 4.34 x 10^-4^ | 8.00 | 87.5 |  | 5.09 x 10^-8^ | 16.00 | 93.8 |
| Esophageal squamous cell carcinoma  ($n_{1}=20,n_{2}=1390$) | 0 | 2.08 x 10^-4^ | 1.00 | 0.0 |  | 2.94 x 10^-9^ | 1.00 | 0.0 |  | 4.14 x 10^-14^ | 1.00 | 0.0 |
|  | 2 x 10^-9^ | 3.00 x 10^-4^ | 1.44 | 30.6 |  | 5.08 x 10^-9^ | 1.73 | 42.1 |  | 8.59 x 10^-14^ | 2.07 | 51.8 |
|  | 5 x 10^-9^ | 4.69 x 10^-4^ | 2.25 | 55.6 |  | 9.92 x 10^-9^ | 3.37 | 70.4 |  | 2.10 x 10^-13^ | 5.06 | 80.2 |
|  | 1 x 10^-8^ | 8.33 x 10^-4^ | 4.00 | 75.0 |  | 2.35 x 10^-8^ | 8.00 | 87.5 |  | 6.63 x 10^-13^ | 16.00 | 93.8 |
| Lung adenocarcinoma  ($n_{1}=30,n_{2}=6$) | 0 | 1.39 x 10^-4^ | 1.00 | 0.0 |  | 5.01 x 10^-11^ | 1.00 | 0.0 |  | 1.80 x 10^-17^ | 1.00 | 0.0 |
|  | 2 x 10^-9^ | 2.00 x 10^-4^ | 1.44 | 30.6 |  | 8.66 x 10^-11^ | 1.73 | 42.1 |  | 3.74 x 10^-17^ | 2.07 | 51.8 |
|  | 5 x 10^-9^ | 3.13 x 10^-4^ | 2.25 | 55.6 |  | 1.69 x 10^-10^ | 3.37 | 70.4 |  | 9.13 x 10^-17^ | 5.06 | 80.2 |
|  | 1 x 10^-8^ | 5.56 x 10^-4^ | 4.00 | 75.0 |  | 4.01 x 10^-10^ | 8.00 | 87.5 |  | 2.89 x 10^-16^ | 16.00 | 93.8 |
| Osteosarcoma  ($n_{1}=22,n_{2}=5$) | 0 | 3.06 x 10^-7^ | 1.00 | 0.0 |  | 8.26 x 10^-14^ | 1.00 | 0.0 |  | 2.23 x 10^-20^ | 1.00 | 0.0 |
|  | 2 x 10^-9^ | 4.40 x 10^-7^ | 1.44 | 30.6 |  | 1.43 x 10^-13^ | 1.73 | 42.1 |  | 4.62 x 10^-20^ | 2.07 | 51.8 |
|  | 5 x 10^-9^ | 6.88 x 10^-7^ | 2.25 | 55.6 |  | 2.79 x 10^-13^ | 3.37 | 70.4 |  | 1.13 x 10^-19^ | 5.06 | 80.2 |
|  | 1 x 10^-8^ | 1.22 x 10^-6^ | 4.00 | 75.0 |  | 6.60 x 10^-13^ | 8.00 | 87.5 |  | 3.57 x 10^-19^ | 16.00 | 93.8 |
| Thyroid papillary/follicular carcinoma  ($n_{1}=26,n_{2}=7$) | 0 | 7.31 x 10^-6^ | 1.00 | 0.0 |  | 2.41 x 10^-12^ | 1.00 | 0.0 |  | 7.96 x 10^-19^ | 1.00 | 0.0 |
|  | 2 x 10^-9^ | 1.05 x 10^-5^ | 1.44 | 30.6 |  | 4.17 x 10^-12^ | 1.73 | 42.1 |  | 1.65 x 10^-18^ | 2.07 | 51.8 |
|  | 5 x 10^-9^ | 1.64 x 10^-5^ | 2.25 | 55.6 |  | 8.14 x 10^-12^ | 3.37 | 70.4 |  | 4.03 x 10^-18^ | 5.06 | 80.2 |
|  | 1 x 10^-8^ | 2.92 x 10^-5^ | 4.00 | 75.0 |  | 1.93 x 10^-11^ | 8.00 | 87.5 |  | 1.27 x 10^-17^ | 16.00 | 93.8 |

Table A4 in S1 Appendix. Linear regression analysis of REIC for Japanese population [2] (using ICRP recommended weighting [3] of EAR vs ERR risks) (dependent variable) versus probability[at least one mutation is mutagen-induced | cancer occurs] (independent variable). The conditional probability is evaluated (via expression (9)) using a generalization of the model of Wu *et al.* [1] using *k* = 1 to 4 critical cancer mutations, a spontaneous mutation rate of $u_{S}$ =10^-8^ per cell division, and a mutagen-induced mutation rate, $u_{M}$= 2 x 10^-9^, 5 x 10^-9^ or 1 x 10^-8^ per cell division, mutagen-associated rates increase from 0 after the first third of stem cell divisions. The data used in the regression are given in Table A1 in S1 Appendix and in Table 1 of Little *et al.* [4].

| Number of cancer mutations *k* | Spontaneous mutation rate ($u_{S}$) | Mutagen-induced mutation rate ($u_{M}$) | *p*-value of trend / *p*-value of trend with outliers removed^a^ | R^2^ | Pearson correlation coefficient | Spearman correlation coefficient |
| --- | --- | --- | --- | --- | --- | --- |
| 1 | 1 x 10^-8^ | 2 x 10^-9^ | 0.8814 / 0.8814 | 0.0030 | 0.2196 | 0.1553 |
| 2 | 1 x 10^-8^ | 2 x 10^-9^ | 0.3059 / 0.6670 | 0.1301 | 0.0211 | 0.0732 |
| 3 | 1 x 10^-8^ | 2 x 10^-9^ | 0.4968 / 0.8178 | 0.0596 | -0.0963 | -0.0549 |
| 4 | 1 x 10^-8^ | 2 x 10^-9^ | 0.4970 / 0.8167 | 0.0595 | -0.0964 | -0.0686 |
| 1 | 1 x 10^-8^ | 5 x 10^-9^ | 0.9694 / 0.9694 | 0.0002 | 0.2320 | 0.1553 |
| 2 | 1 x 10^-8^ | 5 x 10^-9^ | 0.3189 / 0.7101 | 0.1237 | 0.0254 | 0.1075 |
| 3 | 1 x 10^-8^ | 5 x 10^-9^ | 0.4965 / 0.8159 | 0.0597 | -0.0959 | -0.0549 |
| 4 | 1 x 10^-8^ | 5 x 10^-9^ | 0.4969 / 0.8138 | 0.0595 | -0.0959 | -0.0549 |
| 1 | 1 x 10^-8^ | 1 x 10^-8^ | 0.9749 / 0.9749 | 0.0001 | 0.2154 | 0.0850 |
| 2 | 1 x 10^-8^ | 1 x 10^-8^ | 0.3376 / 0.7627 | 0.1151 | 0.0328 | 0.1219 |
| 3 | 1 x 10^-8^ | 1 x 10^-8^ | 0.4961 / 0.8141 | 0.0598 | -0.0953 | -0.0549 |
| 4 | 1 x 10^-8^ | 1 x 10^-8^ | 0.4970 / 0.8102 | 0.0595 | -0.0950 | -0.0549 |

^a^*p*-value of trend with all high-leverage datapoints, with Cook’s-distance > 4/[*n* – *p* - 1] (*n*=number of datapoints, *p*=number of fitted parameters), removed.

Table A5 in S1 Appendix. Linear regression analysis of REIC for Japanese population [2] (using BEIR VII recommended weighting [5] of EAR vs ERR risks) (dependent variable) versus probability[at least one mutation is mutagen-induced | cancer occurs] (independent variable). The conditional probability is evaluated (via expression (9)) using a generalization of the model of Wu *et al.* [1] using *k* = 1 to 4 critical cancer mutations, a spontaneous mutation rate of $u_{S}$=10^-8^ per cell division, and a mutagen-induced mutation rate, $u_{M}$= 2 x 10^-9^, 5 x 10^-9^ or 1 x 10^-8^ per cell division, mutagen-associated rates increase from 0 after the first third of stem cell divisions. The data used in the regression are given in Table A1 of S1 Appendix and in Table 1 of Little *et al.* [4].

| Number of cancer mutations *k* | Spontaneous mutation rate ($u_{S}$) | Mutagen-induced mutation rate ($u_{M}$) | ***p*-value of trend / *p*-value of trend with outliers removed^a^** | R^2^ | Pearson correlation coefficient | Spearman correlation coefficient |
| --- | --- | --- | --- | --- | --- | --- |
| 1 | 1 x 10^-8^ | 2 x 10^-9^ | 0.8721 / 0.4272 | 0.0034 | 0.2196 | 0.1372 |
| 2 | 1 x 10^-8^ | 2 x 10^-9^ | 0.3196 / 0.6758 | 0.1234 | 0.0211 | 0.0764 |
| 3 | 1 x 10^-8^ | 2 x 10^-9^ | 0.3726 / 0.3726 | 0.1003 | -0.0963 | -0.0410 |
| 4 | 1 x 10^-8^ | 2 x 10^-9^ | 0.3729 / 0.3729 | 0.1002 | -0.0964 | -0.0547 |
| 1 | 1 x 10^-8^ | 5 x 10^-9^ | 0.7971 / 0.3921 | 0.0088 | 0.2320 | 0.1372 |
| 2 | 1 x 10^-8^ | 5 x 10^-9^ | 0.3359 / 0.7704 | 0.1159 | 0.0254 | 0.1097 |
| 3 | 1 x 10^-8^ | 5 x 10^-9^ | 0.3726 / 0.3726 | 0.1003 | -0.0959 | -0.0410 |
| 4 | 1 x 10^-8^ | 5 x 10^-9^ | 0.3731 / 0.3731 | 0.1001 | -0.0959 | -0.0410 |
| 1 | 1 x 10^-8^ | 1 x 10^-8^ | 0.7929 / 0.3904 | 0.0091 | 0.2154 | 0.0676 |
| 2 | 1 x 10^-8^ | 1 x 10^-8^ | 0.3595 / 0.8787 | 0.1057 | 0.0328 | 0.1236 |
| 3 | 1 x 10^-8^ | 1 x 10^-8^ | 0.3726 / 0.3726 | 0.1003 | -0.0953 | -0.0410 |
| 4 | 1 x 10^-8^ | 1 x 10^-8^ | 0.3736 / 0.3736 | 0.0999 | -0.0950 | -0.0410 |

^a^*p*-value of trend with all high-leverage datapoints, with Cook’s-distance > 4/[*n* – *p* - 1] (*n*=number of datapoints, *p*=number of fitted parameters), removed.

Table A6 in S1 Appendix. Probability of cancer and relative risk using generalized multistage model with *k*=2 mutations for stem cells and *k*=3 mutations for transit cells, or with *k*=4 mutations for stem cells and *k*=4 mutations for transit cells, allowing for mutations in stem cell and transit cell compartments. This is a special case of the fully-stochastic destabilization model developed by Little *et al.* [6].

|  | Stem cell mutation rate | Transit cell mutation rate | Total probability of cancer | Relative risk | Total probability of cancer | Relative risk |
| --- | --- | --- | --- | --- | --- | --- |
|  |  |  | *k*=2 / *k*=3 | | *k*=3 / *k*=4 | |
| Acute myeloid leukemia (*n*_1_=27, *n*_2_=960) | 1.00 x 10^-8^ | 1.00 x 10^-6^ | 2.85 x 10^-1^ | 1 | 7.58 x 10^-5^ | 1 |
|  | 1.20 x 10^-8^ | 1.20 x 10^-6^ | 3.87 x 10^-1^ | 1.35 | 1.42 x 10^-4^ | 1.88 |
|  | 1.50 x 10^-8^ | 1.50 x 10^-6^ | 5.29 x 10^-1^ | 1.85 | 2.90 x 10^-4^ | 3.83 |
|  | 2.00 x 10^-8^ | 2.00 x 10^-6^ | 7.09 x 10^-1^ | 2.48 | 8.09 x 10^-4^ | 10.7 |
| Basal cell carcinoma (*n*_1_=32, *n*_2_=608) | 1.00 x 10^-8^ | 1.00 x 10^-6^ | 7.67 x 10^-1^ | 1 | 3.09 x 10^-4^ | 1 |
|  | 1.20 x 10^-8^ | 1.20 x 10^-6^ | 8.39 x 10^-1^ | 1.09 | 5.76 x 10^-4^ | 1.86 |
|  | 1.50 x 10^-8^ | 1.50 x 10^-6^ | 9.03 x 10^-1^ | 1.18 | 1.67 x 10^-3^ | 5.39 |
|  | 2.00 x 10^-8^ | 2.00 x 10^-6^ | 9.53 x 10^-1^ | 1.24 | 4.09 x 10^-3^ | 13.2 |
| Colorectal adenocarcinoma (*n*_1_=28, *n*_2_=5840) | 1.00 x 10^-8^ | 1.00 x 10^-6^ | 9.95 x 10^-1^ | 1 | 1.73 x 10^-1^ | 1 |
|  | 1.20 x 10^-8^ | 1.20 x 10^-6^ | 9.97 x 10^-1^ | 1.00 | 2.75 x 10^-1^ | 1.59 |
|  | 1.50 x 10^-8^ | 1.50 x 10^-6^ | 9.98 x 10^-1^ | 1.00 | 4.48 x 10^-1^ | 2.59 |
|  | 2.00 x 10^-8^ | 2.00 x 10^-6^ | 9.99 x 10^-1^ | 1.00 | 6.93 x 10^-1^ | 4.01 |
| Esophageal squamous cell carcinoma (*n*_1_=20, *n*_2_=1390) | 1.00 x 10^-8^ | 1.00 x 10^-6^ | 9.33 x 10^-3^ | 1 | 2.61 x 10^-6^ | 1 |
|  | 1.20 x 10^-8^ | 1.20 x 10^-6^ | 1.46 x 10^-2^ | 1.57 | 4.73 x 10^-6^ | 1.81 |
|  | 1.50 x 10^-8^ | 1.50 x 10^-6^ | 2.58 x 10^-2^ | 2.77 | 1.01 x 10^-5^ | 3.88 |
|  | 2.00 x 10^-8^ | 2.00 x 10^-6^ | 5.42 x 10^-2^ | 5.81 | 2.81 x 10^-5^ | 10.8 |
| Lung carcinoma (*n*_1_=30, *n*_2_=6) | 1.00 x 10^-8^ | 1.00 x 10^-6^ | 2.05 x 10^-6^ | 1 | -^a^ | -^a^ |
|  | 1.20 x 10^-8^ | 1.20 x 10^-6^ | 2.75 x 10^-6^ | 1.34 | -^a^ | -^a^ |
|  | 1.50 x 10^-8^ | 1.50 x 10^-6^ | 3.17 x 10^-6^ | 1.55 | -^a^ | -^a^ |
|  | 2.00 x 10^-8^ | 2.00 x 10^-6^ | 5.00 x 10^-6^ | 2.44 | -^a^ | -^a^ |
| Osteosarcoma (*n*_1_=22, *n*_2_=5) | 1.00 x 10^-8^ | 1.00 x 10^-6^ | 6.18 x 10^-9^ | 1 | -^a^ | -^a^ |
|  | 1.20 x 10^-8^ | 1.20 x 10^-6^ | 7.70 x 10^-9^ | 1.24 | -^a^ | -^a^ |
|  | 1.50 x 10^-8^ | 1.50 x 10^-6^ | 1.18 x 10^-8^ | 1.92 | -^a^ | -^a^ |
|  | 2.00 x 10^-8^ | 2.00 x 10^-6^ | 1.31 x 10^-8^ | 2.12 | -^a^ | -^a^ |
| Thyroid papillary/follicular carcinoma (*n*_1_=26, *n*_2_=7) | 1.00 x 10^-8^ | 1.00 x 10^-6^ | 1.35 x 10^-7^ | 1 | -^a^ | -^a^ |
|  | 1.20 x 10^-8^ | 1.20 x 10^-6^ | 2.09 x 10^-7^ | 1.55 | -^a^ | -^a^ |
|  | 1.50 x 10^-8^ | 1.50 x 10^-6^ | 2.31 x 10^-7^ | 1.71 | -^a^ | -^a^ |
|  | 2.00 x 10^-8^ | 2.00 x 10^-6^ | 4.15 x 10^-7^ | 3.08 | -^a^ | -^a^ |

^a^non-convergence of likelihood integration

# References

1. Wu S, Powers S, Zhu W, Hannun YA. Substantial contribution of extrinsic risk factors to cancer development. Nature. 2016;529(7584):43-7. doi: 10.1038/nature16166. PubMed PMID: 26675728.

2. United Nations Scientific Committee on the Effects of Atomic Radiation (UNSCEAR). UNSCEAR 2006 Report. Annex A. Epidemiological Studies of Radiation and Cancer. New York: United Nations; 2008. p. 13-322.

3. International Commission on Radiological Protection. The 2007 Recommendations of the International Commission on Radiological Protection. ICRP publication 103. Ann ICRP. 2007;37(2-4):1-332. doi: S0146-6453(07)00031-0 [pii];10.1016/j.icrp.2007.10.003 [doi].

4. Little MP, Hendry JH, Puskin JS. Lack of correlation between stem-cell proliferation and radiation- or smoking-associated cancer risk. PloS one. 2016;11(3):e0150335. doi: 10.1371/journal.pone.0150335.

5. Committee to Assess Health Risks from Exposure to Low Levels of Ionizing Radiation NRC. Health Risks from Exposure to Low Levels of Ionizing Radiation: BEIR VII - Phase 2. Washington, DC, USA: National Academy Press; 2006. 1-406 p.

6. Little MP, Kleinerman RA, Stiller CA, Li G, Kroll ME, Murphy MFG. Analysis of retinoblastoma age incidence data using a fully stochastic cancer model. Int J Cancer. 2012;130(3):631-40. doi: 10.1002/ijc.26039. PubMed PMID: 21387305; PubMed Central PMCID: PMC3167952.

**Supporting Information Legends**

WinZip archive containing data, R scripts and output files, Excel spreadsheets, and Fortran code (*.for) and associated input (*.inp) and output (*.lis) files (“Archive for PLoS Comput Biol paper.zip”)
